# Time-dependent structural transformation analysis to high-level Petri net model with active state transition diagram

**DOI:** 10.1186/1752-0509-4-39

**Published:** 2010-04-01

**Authors:** Chen Li, Masao Nagasaki, Ayumu Saito, Satoru Miyano

**Affiliations:** 1 Human Genome Center, Institute of Medical Science, University of Tokyo, 4-6-1 Shirokanedai, Minato-ku, Tokyo 108-8639, Japan

## Abstract

**Background:**

With an accumulation of *in silico *data obtained by simulating large-scale biological networks, a new interest of research is emerging for elucidating how living organism functions over time in cells.

Investigating the dynamic features of current computational models promises a deeper understanding of complex cellular processes. This leads us to develop a method that utilizes structural properties of the model over all simulation time steps. Further, user-friendly overviews of dynamic behaviors can be considered to provide a great help in understanding the variations of system mechanisms.

**Results:**

We propose a novel method for constructing and analyzing a so-called *active state transition diagram *(ASTD) by using time-course simulation data of a high-level Petri net. Our method includes two new algorithms. The first algorithm extracts a series of subnets (called *temporal subnets*) reflecting biological components contributing to the dynamics, while retaining positive mathematical qualities. The second one creates an ASTD composed of unique temporal subnets. ASTD provides users with concise information allowing them to grasp and trace how a key regulatory subnet and/or a network changes with time. The applicability of our method is demonstrated by the analysis of the underlying model for circadian rhythms in *Drosophila*.

**Conclusions:**

Building ASTD is a useful means to convert a hybrid model dealing with discrete, continuous and more complicated events to finite time-dependent states. Based on ASTD, various analytical approaches can be applied to obtain new insights into not only systematic mechanisms but also dynamics.

## Background

A great deal of biological datasets have been measured in a lot of laboratories around the world in recent years. Petri nets have been applied successfully in modeling, simulating and analyzing biological networks [1,2] (i.e., metabolic [3,4], signal transduction [5,6] and gene regulatory networks [7,8]). In the meanwhile, a number of public and commercial databases have developed tools to automatically convert biological pathway information into various formats of models, e.g., the tool TRANSPATH2CSML [9] automatically converts data stored in TRANSPATH [10] to a simulation-based model encoded in a biological pathway format. These approaches make it possible to construct larger and more complex biological network models. However, the associated increase in complexity and output data result in the difficulty of grasping systematic characteristics of the models.

Several studies with respect to the topology of the interactions between biological compounds in cellular networks based on Petri net theory have been made in understanding biological networks [11-14]. These approaches use mathematical properties of Petri nets (e.g., reachability, liveness, boundedness and T-invariant) to reveal some topological properties of biological networks on qualitative models. Other investigation regarding the dynamics of signal propagation in signaling pathway has been given by Hardy *et al. *[15]. This method gives temporal information about the flow of signal propagation. However, the analysis is limited to a single signal source. Nevertheless, it is expected to find a general methodology to analyze the dynamics with quantitative simulation information, i.e., time-dependent dynamic behaviors among genes and their products which constitute biological networks [16].

This paper presents a novel method to build a framework for automatically constructing a so-called *active state transition diagram *(ASTD) for the dynamic analysis with respect to the structural changes over time in a *hybrid functional Petri net with extension *(HFPNe) model. Our method incorporates time-course simulation data and temporal structural properties (connection relationship) of the HFPNe model. This method constructs an ASTD composed of unique temporal subnets which are exhaustively extracted from original HFPNe model, produces a simplified graphical representation about the temporal information of the dynamics. After the ASTD is built, various analysis can be applied to the ASTD to obtain new insights into the systematic dynamics. Note that HFPNe is an enhanced Petri net architecture which involves the functions of existing high-level Petri nets [17].

The paper is organized as follows. In Methods, we first present a basic definition of HFPNe. We propose two new algorithms for constructing ASTD based on time-course simulation data from an HFPNe model. In Results and Discussion, we present a case study describing how our method is employed to integrate and interpret the circadian rhythm model in *Drosophila*, and give three characteristic overviews of the ASTD for facilitating a system-level understanding. The final section concludes our paper and addresses the contribution.

## Methods

### Hybrid functional Petri net with extension (HFPNe)

**Figure 1:**
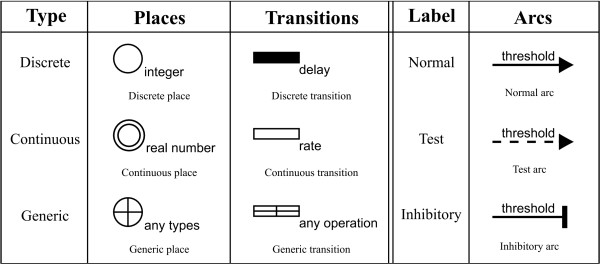
**The symbols of HFPNe**.

Hybrid functional Petri net with extension (HFPNe) is a mathematical tool for modeling and simulating biological networks. HFPNe can deal with three types of data - discrete, continuous and generic - and is comprised of three types of elements - *places, transitions *and *arcs *- whose symbols are illustrated in Figure 1.

A discrete place holds a positive integer number of content. A discrete transition is the same notion as used in the traditional discrete Petri net [18]. A continuous place holds a nonnegative real number as concentration of a substance such as mRNA and protein. A continuous transition is used to represent a biological reaction such as transcription and translation, at which the reaction speed is assigned as a parameter. A generic place can hold any kind of types including object, e.g., the string of nucleotide base sequence. A generic transition can deal with any kind of operations (e.g., alternative splicing and frameshifting) to all types of places. Generic place and transition have been practically applied for modeling and simulating more complicated biological processes [7,19,20], e.g., activities of enzymes for a multi-modification protein. Arcs are classified into three types: normal arc, test arc, and inhibitory arc. Normal arc connects a place to a transition or vice versa. Test or inhibitory arc represents a condition and is only directed from a place to a transition. Each of normal arc from a place, test arc, and inhibitory arc has a threshold by which the parameter assigned to the transition at its head is controlled. A normal arc from a place or a test arc (an inhibitory arc) can participate in activating (repressing) a transition at its head, as far as the content of a place at its tail is over the threshold. For either of test or inhibitory arcs, no amount is consumed from a place at its tail.

### Basic definitions for HFPNe

We briefly give the necessary definitions for HFPNe used in this paper. The formal definition of HFPNe is given as additional material [see Additional file 1]. For further definition and application of HFPNe the reader is suggested to refer to Nagasaki *et al. *[17]. The following is the mathematic definitions used in this paper:

**Definition 1**. A *hybrid functional Petri net with extension *(HFPNe) *H *= (*P*, *T*, *A*, *τ*, *w*, *u*, *d*) consists of the following:

1. *P *is a set of *places *and *T *is a set of *transitions*. Place is labeled with either discrete, continuous, or generic. Transition is also labeled with discrete, continuous, or generic. The place and transition are called *discrete, continuous*, or *generic *according to its label.

For each transition *t *in *T*, it has two sets *Input*_*t *_and *Output*_*t *_of arcs. Arc *a*∈*Input*_*t *_is an edge from input place *p*_*a *_to the transition *t *called *input arc*. Arc *a'*∈*Output*_*t *_is an edge from the transition *t *to output place *p*_*a' *_called *output arc*. Each arc is labeled with either normal, test, or inhibitory, and arc labeled with normal (resp., test, inhibitory) is called *normal arc *(resp., *test arc, inhibitory arc*). We also say that arcs (*a *and *a'*) are *discrete *(resp., *continuous, generic*) if transition *t *is discrete (resp., continuous, generic).

We denote by *PT *and *TP *the set of input arcs and the set of output arcs of all transitions, respectively. We also denote arc *a *in *PT *as *a*(*p*, *t*). In a similar way, arc *a' *in *TP *is denoted as *a'*(*t*, *p*). The set *A *of arcs is given by *PT*∪*TP*.

2. The types of places are given by a type function *τ*.

3. For each input arc *a*∈*PT*, its *activity w*(*a*) is given by an *activity function w*. Activity function *w*(*a*) is used as a function giving the threshold in discrete and continuous cases and the condition in generic case, which is required for enabling the transition *t*.

4. For each arc *c *(*c *= *a*(*p*, *t*)∈*PT *or *c *= *a'*(*t*, *p*)∈*TP*), the *update u*(*c*) is given by an *update function u*.

5. For each discrete or generic transition *t*, the *delay *of *t *is given by a *delay function d*.

We use the parameter *x *≥ 0 for the *time *in HFPNe. Do not confuse *t *for transition with *x *for time.

A *marking *of *P *is defined as a mapping *M *that assigns a mark (the type of contents) to each place. *M *[*p*] is called the *mark *of *p*. The *initial marking I *is a marking at time *x *= 0 and we denote the *marking at time x *by *M*(*x*). The *reserved marking M*_*r*_(*x*) at time *x *represents the amount of "tokens" reserved for firing when firing conditions are satisfied. By convention, let *M*(*p*, *x*) be *M *[*p*](*x*), and *M*_*r*_(*p*, *x*) be *M*_*r *_[*p*](*x*) for *p*∈*P*. We define  (*x*) by  [*p*](*x*) = *M *[*p*](*x*)-*M*_*r *_[*p*](*x*) if *p *is discrete or continuous and  [*p*](*x*) = *M *[*p*](*x*) if *p *is generic. Given the initial marking of HFPNe, the marking *M*(*x*) and the reserved marking *M*_*r*_(*x*) at time *x *are defined in the following way:

For time *x *= 0, *M*(0) = *I *by definition. We define *M*_*r *_[*p*](0) = 0 if *p *is discrete or continuous, and *M*_*r *_[*p*](0) = null (the empty list) if *p *is generic. For *x>*0, we define *M *(*x*) and *M*_*r*_(*x*) in the following way. For transition *t *at time *x*, we say that *t *is *enabled at time x *if the following conditions are satisfied. Otherwise the transition is said to be *disabled at time x*.

1. If *t *is discrete or continuous, then for all input arcs *c *= *a*(*p*, *t*)∈*PT *the following conditions hold:

(a)  [*p*](*x*)*>w*(*c*) [*M*(*x*)] if *a *is not labeled with inhibitory;

(b)  [*p*](*x*)*<w*(*c*) [*M*(*x*)] if *a *is labeled with inhibitory,

where *w*(*c*) [*M*(*x*)] is the threshold value of *c *on marking *M *at time *x*.

2. If *t *is generic, then for all input arcs *a*(*p*, *t*)∈*PT *the following conditions hold:

(a) *w*(*a*) [(*x*)] = true if *a *is not labeled with inhibitory;

(b) *w*(*a*) [(*x*)] = false if *a *is labeled with inhibitory.   □

**Definition 2**. For arc *c *= *a*(*p*, *t*)∈*PT *at time *x*, we say that *c *is *enabled at time x *if the following conditions are satisfied. Otherwise, the arc *c *is said to be *disabled at time x*.

1. If *c *is discrete or continuous, then  [*p*](*x*)*>w*(*c*) [*M*(*x*)] holds;

2. If *c *is generic, then *w*(*c*) [(*x*)] = true holds.   □

**Definition 3**. If disabled transition *t *turns enabled at time *x*, we say that *t *is *triggered at time x *and *x *is called the *trigger time*. If enabled transition *t *turns disabled at time *x*, we say that *t *is *switched off at time x *and *x *is called the *switch-off time*.   □

**Definition 4**. We define *firing *of discrete transition *t*. Assume that discrete transition *t *is triggered at time *x*. For each normal input arc *a*(*p*, *t*), the place *p *must be discrete or continuous by definition. Then *M*_*r *_[*p*] reserves *a · u*(*a*) [*M *(*x*)], i.e., *α*·*u*(*a*) [*M *(*x*)] is added to *M*_*r *_[*p*], for the time *y > x *until *x *+ *d*(*t*) [*M*(*x*)], where *α *= {0, 1}, if *α *= 0, reserve is disabled; otherwise, token is reserved. If *t *is still enabled at *x *+ *d*(*t*) [*M*(*x*)], then at the same time *x *+ *d*(*t*) [*M*(*x*)], *M *[*p*] is decreased by *u*(*a*) [*M*(*x*)] and *M*_*r *_[*p*] releases *u*(*a*) [*M *(*x*)], i.e., *u*(*a*) [*M *(*x*)] is decreased from *M*_*r *_[*p*]. Simultaneously, for each output normal arc *a'*(*t*, *p'*), *M *[*p'*] is increased by *u*(*a'*) [*M*(*x*)] at time *x *+ *d*(*t*) [*M*(*x*)] by arc *a'*(*t*, *p'*). The time *d*(*t*) [*M*(*x*)] is called the *delay *that is determined by the function *d*(*t*) of the mark *M*(*x*) at time *x*.

As we will describe in Definitions 5 and 6 below, the reservation is not performed by generic or continuous transition. However, for the place *p*, there may be another discrete transitions *t*_1_,..., *t*_ℓ _with normal input arcs *a*_1_(*p*, *t*_1_),..., *a*_*m*_(*p*, *t*_ℓ_) which are triggered at time *x*. Then each discrete transition *t*_*i *_tries to reserve *u*(*a*_*i*_) [*M *(*x*)] from the same *M *[*p*] at time *x *for *i *= 0,..., ℓ, where *a*_0 _= *a*(*p*, *t*) and *t*_0 _= *t*. We say that there is a *conflict *with *p *at time *x *if . When a conflict occurs, some *conflict resolution *should be applied, e.g., random selection of transitions, priorities on transitions, etc.

Even if some conflict resolution procedure selected the transition *t *to go further, the place *p *of *a*(*p*, *t*) may be input places or output places of another discrete/continuous/generic transitions. By this, *M *[*p*] and *M*_*r *_[*p*], and therefore  [*p*], may be changed, the conditions of "enabled" are not be necessarily satisfied until the firing time *x *+ *d*(*t*) [*M*(*x*)]. When *t *becomes disabled before *x *+ *d*(*t*) [*M*(*x*)], we say that a *system error *occurs with *t*.

Thus triggered transition does not necessarily fire. If all of these actions succeed, we say that *t fires *at time *x *+ *d*(*t*) [*M*(*x*)].   □

Note that *α *is set to zero in this paper so that the reserved marking *M*_*r*_(*x*) at any time *x *equals to zero. That is, the token amount will not be reserved during the delay time of the transition *t *when it becomes enabled.

**Definition 5**. We define *firing *of generic transition *t*. Assume that generic transition *t *is triggered at time *x*. For each normal input arc *a*(*p*, *t*), the place *p *can be discrete, continuous and generic. For each output normal arc *a'*(*t*, *p'*), *p' *can be also any kind of places. If *t *keeps enabled until time *x *+ *d*(*t*) [*M*(*x*)], then *M *[*p*] at time *x *+ *d*(*t*) [*M*(*x*)] is updated to *u*(*a*) [*M*(*x*)] and *M *[*p'*] is updated to *u*(*a'*) [*M*(*x*)] at time *x *+ *d*(*t*) [*M*(*x*)]. We say that *t fires *at time *x *+ *d*(*t*) [*M*(*x*)] if this action succeeds. If *p *is generic, it is always that *M*_*r *_[*p*](*x*) = null. No change is added to *M*_*r *_[*p*] by arc *a*(*p*, *t*) if *p *is discrete or continuous. In a similar way to discrete transition, if *p *is discrete or continuous, *Mp *and *M*_*r *_[*p*] have a possibility to be changed before *x *+ *d*(*t*) [*M*(*x*)] by another transitions. Therefore *w*(*a*)[(*y*)] = true is not necessarily kept for *y *∈ (*x*, *x *+ *d*(*t*) [*M*(*x*)]). As in the case of discrete transition, it should be reported as system error. Since generic transition updates *M *[*p*] and *M *[*p'*] at time *x *+ *d*(*t*) [*M*(*x*)], there is a possibility of conflict with another transitions which use *p *and *p'*. Thus some conflict resolution should be applied or it should be reported as system error.   □

**Definition 6**. We define *firing *of continuous transition *t*. When continuous transition *t *is triggered, it starts firing and updates the marks of its connected places continuously with the speeds determined by the update function *u *and the marking *M *as long as it is enabled. Assume that continuous transition *t *is enabled at time *x*. For each normal input arc *a*(*p*, *t*), the place *p *must be continuous by definition. Then the mark *M *[*p*] will be decreased through the arc *a*(*p*, *t*) with the additional speed *u*(*a*) [*M*(*x*)] at time *x*. No change is added to *M*_*r *_[*p*] by arc *a*(*p*, *t*). For output normal arc *a'*(*t*, *p'*), the place *p' *must be continuous by definition. Then the mark *M *[*p'*] will be increased through the arc *a'*(*t*, *p'*) with the additional speed *u*(*a'*) [*M*(*x*)] at time *x*. No change is added to *M*_*r *_[*p'*] by arc *a'*(*t*, *p'*).   □

### HFPNe modeling

• *Places *are used to model biological molecules, conditions, states and cellular organelles. In the case of chemical reactions, the compounds involved usually have specific quantities. In HFPNe, places can take any object that can be expressed in programming languages like an instance of a class in C++ or Java.

• *Transitions *are used to model interactions among places, such as phosphorylation, translocation, and apoptosis. In HFPNe, each transition can define any event/function that can be performed by programming languages. In a simple model, the event/function can be the speed of a reaction or a discrete reaction.

• *Arcs *connecting the places and the transitions represent the relations between corresponding substances and interactions.

As described above, HFPNe model allows modeling and simulation of biological networks combining both an intuitive graphical representation and well-founded mathematic definition. Because of the versatility of HFPNe, it has been successfully employed to develop and analyze complex biological networks [7,19,20]. For example, in [7], Li *et al. *employed a series of generic places/transitions to realize 48 distinct genetic conditions that are the combination of four genes (*lin12*, *lin15*, *vul *and *lst*) and one anchor cell (AC) for determining the vulval precursor cell fate. AC, *lin15*, *vul *and *lst *can toggle between true and false. *lin12 *has three string-type values, i.e., "wt", "ko", "gf", indicating three genetic conditions of wild, knockout and overexpression of *lin12 *(refer to Figures seven and eight in [7]). Saito *et al. *[19] applied HFPNe to model regulatory networks that involve new key regulator microRNA. They selected the cell fate determination model of two gustatory neurons of *Caenorhabditis elegans *- ASE left (ASEL) and ASE right (ASER) (see Figure three in [19]). These neurons are morphologically bilaterally symmetric but physically asymmetric in function. By the simulation, they have confirmed the hypothesis that the cell fate is determined by the double-negative feedback loop involving *lsy-6 *and *mir-273 *microRNAs. Tasaki *et al. *[20] introduced time-series proteomic data to the HFPNe model. The authors semi-automatically constructed a well-tuned epidermal growth factor receptor signal transduction pathway model (EGFR model, see Figure two in [20]) coupled with their data assimilation (DA) framework.

### Model Analysis

**Figure 2:**
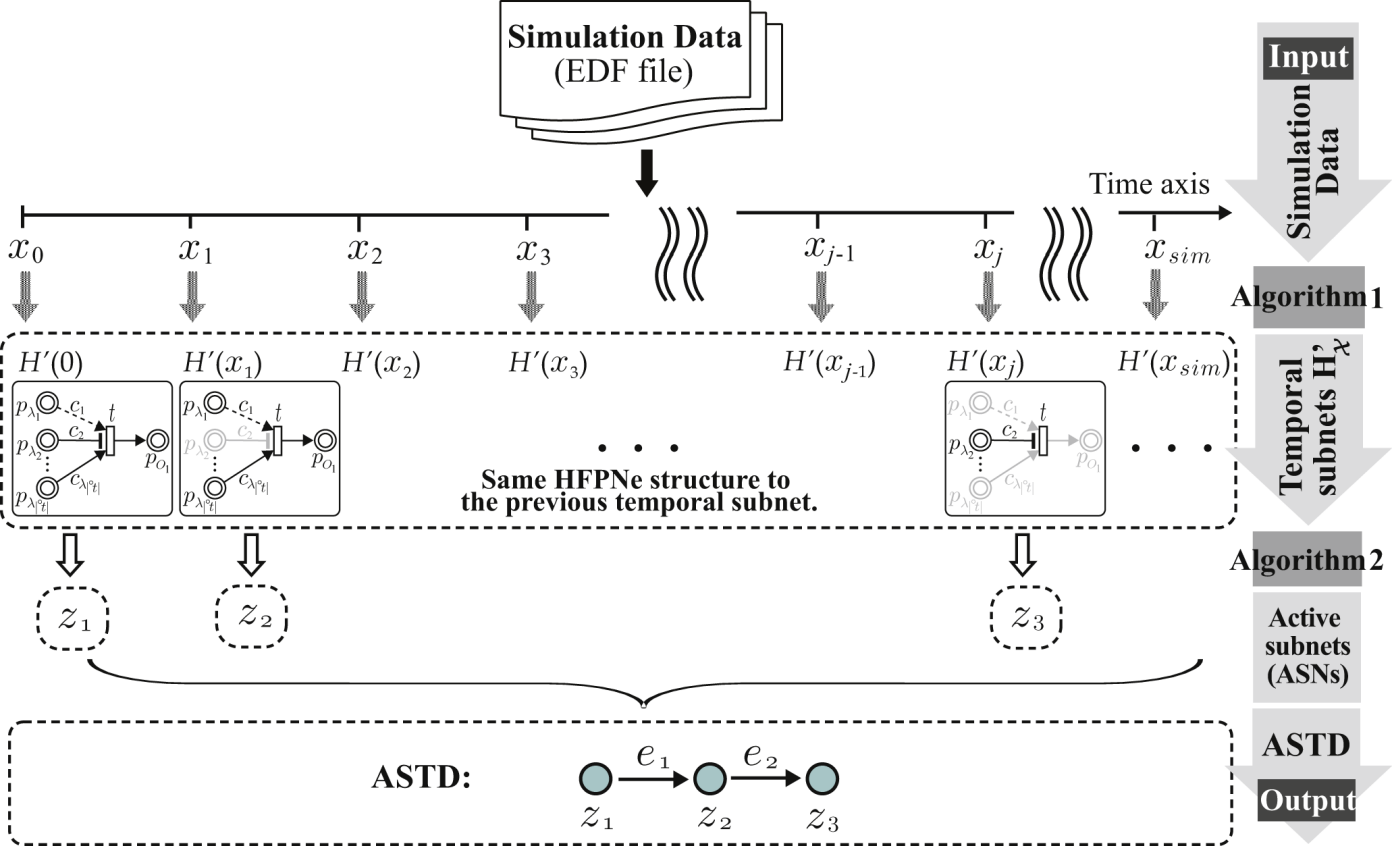
**Schematic overview of our method**. The method includes Algorithm 1 and Algorithm 2 for constructing an ASTD (active state transition diagram) from simulation data (EDF file).

We present two new algorithms (i.e. Algorithm 1 and Algorithm 2) for analyzing structural transformation of HFPNe model over time. Figure 2 illustrates a schematic overview describing how to use Methods for the analysis based on time-course simulation data.

### Algorithm to extract temporal subnet from time-course simulation data

First we show Algorithm 1 for extracting temporal subnets from time-course simulation data (*simulation data *for short). The simulation data is generated by using the simulator of HFPNe, which is saved in an expression data format (called EDF). In EDF, the concentrations of all places (i.e., the marking) are stored at every time point during the simulation (in this case, a constant time interval). Let  = *x*_0_*x*_1_⋯*x*_*sim *_be a non-empty list of simulation time points, where *x*_0 _is a start time point of the simulation and *x*_*sim *_is an end time point. The size (the number of elements) of the list  is denoted by ||.

Algorithm 1 aims to (i) extract a minimal element set of the HFPNe model at time *x *(i.e., the extracted set cannot be reduced furthermore). In other words, such a minimal element set with corresponding concentration distribution *M*(*x*), will return exactly the same simulation results as the original model under a precondition that the elapsed delay time of the discrete transition is given in the EDF. Any disturbance to the elements belonging to this minimal set will lead to different simulation results; and (ii) derive a total minimal element sets by exhaustively examining all reachable states of the HFPNe model with respect to the structural transformations along the time variations. We thus define *temporal subnet H'*(*x*) at time point *x *as such a minimal element set consisting of usable HFPNe elements involving:

(1) enabled arcs;

(2) transitions connected by (1); and

(3) places connected by (1) and places connected from (2).

The temporal subnet *H'*(*x*_*i*_) has following positive mathematical qualities: (i) the places involved in *H'*(*x*_*i*_) is able to be simulated with corresponding concentration distribution *M*(*x*_*i*_), and the simulation result of these places from *x*_*i *_to *x*_*i*+1 _is exactly the same to the one of the original HFPNe model; and (ii) all elements in *H'*(*x*_*i*_) take part in the firing at *x*_*i*_, i.e., corresponding biological components do participate in regulatory activity. We formalize this notion in the following definitions:

**Definition 7**. Let *H *= (*P*, *T*, *A*, *τ*, *w*, *u*, *d*) be an HFPNe.

(1) Let *p *be a place in *P*, °*p *(or *p*°) is a set of the input (or output) transitions of *p*.

(2) *A*^*T *^is a set of the test arcs; *A*^*I *^is a set of the inhibitory arcs; and *A*^*N *^is a set of the normal input arcs.

(3) For each arc *c*, let °*c *denote the source of *c*; let *c*° denote the target of *c*.   □

**Definition 8**. Let *t *be a transition of the given HFPNe *H*.

(1) °*t *is the set of the input places {*pλ*_1_, *pλ*_2_,⋯, *pλ*_|°*t*|_} of *t*; *t*° is the set of the output places {*PO*_1_, *PO*_2_, ⋯, *PO*_|*t*°|_} of *t*.

(2) *PT*^*t *^is the set of the arcs from the places in °*t *to *t*; *TP*^*t *^is the set of the arcs from *t *to the places in *t*°.

(3) The set of input places connected by the inhibitory arcs to the transition *t *is denoted as . *A*^*I*, *t *^is the set of the inhibitory arcs . Similarly, the set of the test (or normal) arcs from the input places of *t *to *t *is denoted as *A*^*T*, *t*^(or *A*^*N*, *t*^).

(4)  is the set of disabled normal and test arcs {*c|*(*c*∈(*A*^*T*, *t*^∪*A*^*N*, *t*^)) ∧ ((*w*(*c*)≥*M *[°*c*])∨(*w*(*c*) = false))}, where *w*(*c*) is the activity function.  is the set of enabled inhibitory arcs {*c|*(*c*∈*A*^*I*, *t*^) ∧ ((*w*(*c*) <*M *[°*c*])∨(*w*(*c*) = true))}.   □

Algorithm 1. EXTRACTING TEMPORALSUBNET

For a given HFPNe *H *= (*P*, *T*, *A*, *τ*, *w*, *u*, *d*) at time *x*, calculate ***TSN***(*H*, *x*) and return *H'*(*x*).

***TSN***(*H*, *x*):

1. *H'*←*H*

2. ***RMVA***(*H'*)      /* delete disabled arcs */

3. ***RMVT***(*H'*)      /* delete isolated transitions */

4. ***RMVP***(*H'*)      /* delete isolated places*/

5. return *H'*

***RMVA***(*H'*):

For ∀*t*∈*T *of *H'*,

1. **if ***|A*^*I*, *t*^*| *= 0      /* if there exists no inhibitory arc */

   /* if there exists such a normal/test arc whose evaluated value of activity function is greater than or equal to the concentration of the connected place */

2.   **if ** delete *PT*^*t*^∪*TP*^*t*^

3. **else**      /* more than one inhibitory arc existing */

4.   **if **

      /* if there exists an inhibitory arc whose evaluated value of activity function is less than the concentration of the connected place */

5.      **if ** delete ∪*TP*^*t*^

6.      **else **delete *PT*^*t*^∪*TP*^*t*^

7.   **else**

8.      **if ** delete ∪*TP*^*t*^

9.      **else **delete *A*^*I*, *t*^

***RMVT***(*H'*):

For ∀*t*∈*T *of *H*,

**if **((*PT*^*t *^= *ϕ*) ∧ (*TP*^*t *^= *ϕ*)) delete *t*   /* delete isolated transition */

***RMVP***(*H'*):

For ∀*p*∈*P *of *H*,

**if **((°*p *= *ϕ*) ∧ (*p*° = *ϕ*)) delete *p*   /* delete isolated place */

**Figure 3:**
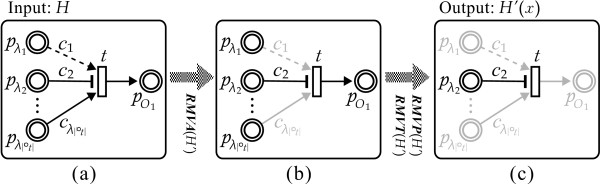
**An example illustrating the process of performing Algorithm 1**. For a given transition *t*, the processes of extracting a temporal subnet *H'*(*x*) from *H *at time *x *by applying **Algorithm 1**. Block (b) is obtained from (a) by performing the subroutine ***RMVA***(*H'*) (step 2 in ***TSN***(*H*, *x*)). Block (c) is obtained from (b) by performing the subroutine ***RMVP***(*H'*) (step 4 in ***TSN***(*H*, *x*)). Note that *t *is connected by an enabled inhibitory arc, therefore *t *cannot be removed by ***RMVT***(*H'*) (step 3 in ***TSN***(*H*, *x*)) in **Algorithm 1**.

The above algorithm ***TSN***(*H*, *x*) is composed of three parts: ***RMVA***(*H'*), ***RMVT***(*H'*) and ***RMVP***(*H'*). ***RMVA***(*H'*) is designed to eliminate disabled arcs. ***RMVT***(*H'*) and ***RMVP***(*H'*) are designed to eliminate isolated transitions and places respectively since such elements cannot participate in regulatory interactions. Figure 3 illustrates the processes of extracting temporal subnet *H'*(*x*) from *H *by applying the above algorithm with a given transition *t*. In Figure 3, inhibitory arc *c*_2 _has an evaluated value *w*(*c*_2_) less than the concentration *M *[°*c*_2_] of its connected place *pλ*_2. _That means (1) the inhibitory arc *c*_2 _represses the activity of transition *t*; and (2) two arcs *c*_1 _and  are disabled due to the inhibition via *c*_2_. These arcs are consequently deleted at step 5 in ***RMVA ***(*H'*) (see the procedure from block (a) to (b)). Further, due to the inhibition from *pλ*_2 _which prevents the token amount from flowing into the place *po*_1_, the output arc *a'*(*t*, *po*_1_) of *t *is thus deleted in step 5. This results in three isolated places *pλ*_1_,  and *po*_1 _which are all deleted at step 4 in ***TSN ***(*H*, *x*) (see the procedure from block (b) to (c) in Figure 3). Time complexity of above algorithm to calculate an *H *for *x *is *O*(*|A|*+*|T|*+*|P|*), where *O*(*|A|*) is the time complexity of ***RMVA ***(*H'*). Likewise, *O*(*|T|*) is the time complexity of ***RMVT***(*H'*) and *O*(*|P|*) is the time complexity of ***RMVP***(*H'*). By repeating the above algorithm to the list of simulation time points  = *x*_0_*x*_1_⋯*x*_*sim*_based on the simulation data EDF, we can obtain a multiset  of all temporal subnets *{H'*(*x*_0_), *H'*(*x*_1_),⋯, *H'*(*x*_*sim*_)}.

### Building Active State Transition Diagram (ASTD)

We here show the other algorithm based on the outputs (i.e., temporal subnets) obtained in Algorithm 1. For a given time points list  and a set of temporal subnets  obtained from Algorithm 1, by applying following Algorithm 2, we derive a directed graph, called *active state transition diagram *(ASTD), which is denoted by *G *= (*N*, *E*). *N *represents a set of distinct nodes *{z*_1_,⋯, *z*_|*N*|_}. Each node *z*∈*N *is called *active subnet (ASN) *(or *state *for short), extracted from the set of temporal subsets  without repetition. *E *denotes a set of directed *edges e *from *z*_*i *_to *z*_*j*_, represented by *e *= (*z*_*i*_, *z*_*j*_).

Algorithm 2. CONSTRUCTING ACTIVESTATETRANSITIONDIAGRAM

*x*: current time point.

*sid *: state ID.

*StateMap*: a state map storing *H'*(*x*) as key and *sid *as value.

*Gen*(*H'*(*x*)): a function to output *sid *by referring to the state map.

*curState*: current state.

*prevState*: previous state.

*sim*: simulation time.

1. *x*←*x*_0_, *sid*←1, *z*_1_←*H' *(0), *prevState*←*H'*(0), *N*←{*z*_1_}

2. insert (*prevState*, 1) to *StateMap*, push *sid *to *FIFO *queue *Q*

3. **for ***i *= 1 to *sim*

4.   *x*←*x*_*i*_, *curState*←*H'*(*x*)

5.      **if **(*curState*≠*prevState*)

6.         **if **({*curState*}∩*N *= *ϕ*)

7.            *sid*++, *z*_*sid *_← *H'*(*x*), *N*←*N*∪{*z*_*sid*_}

            insert (*curState, sid*) to *StateMap *and push *sid *to *Q*

            insert edge e = (*z*_*Gen*(*prevState*)_, *z*_*sid*_) to *E*

8.      **else **push *Gen(curState) *to *Q *and

               insert edge *e *= (*z*_*Gen*(*prevState*)_, *z*_*Gen*(*curState*)_) to *E*

9.      *prevState←curState*

10. return *G *= (*N*, *E*)

In **steps 1 **and **2 **of the above algorithm, we mainly build a state map storing temporal subnet *H' *(*x*) as key and state ID *sid *as value that is used as the suffix of node *z*∈*N*. The state map is employed to find the state ID *sid *by referring to the state map when using the function *Gen*(*H'*(*x*)). We also construct a *FIFO *queue *Q *to store the state ID in turn when reading the temporal subnet *H*(*x'*) along the list of  (see Figure 2).

In **steps 3-9**, the procedures are executed until *x*_*sim *_with a constant time interval to find out the ASNs among the temporal subnets . In step 5, we compare current state with previous state, where current state indicates the temporal subnet *H'*(*x*_*i*_) and previous state indicates the temporal subnet *H' *(*x*_*i*-1_) at the previous time point.

If *H'*(*x*_*i*_) is different from *H'*(*x*_*i*-1_), and a temporal subnet equivalent to *H'*(*x*_*i*_) does not exist in *N*, the current state *H'*(*x*_*i*_) will be treated as a new node *z*_*sid *_and the following procedures will be processed in step 7: (i) *N*←*N*∪*{z*_*sid*_}, (ii) insert (current state, state ID) to the state map *StateMap *and push *sid *to *Q*, and (iii) an edge *e*(*H'*(*x*_*i*-1_), *H'*(*x*_*i*_)) from previous state to current state is inserted to *E*. Otherwise if current state *H'*(*x*) already exists in *N*, we (i) push *sid *to *Q*, where *sid *is derived by referring to the state map via the function *Gen*(*H' *(*x*_*i*_)), and (ii) insert an edge from the previous state to the current state to *E*. Time complexity of Algorithm 2 is .

In Figure 2, we can finally derive the ASTD *G *after performing **Algorithm 2**, where *N *= *{z*_1_, *z*_2_, *z*_3_} and *E *= *{e*_1_, *e*_2_}. Three distinct states (i.e., ASNs) *z*_1_, *z*_2_, *z*_3 _are derived from || temporal subnets. It can be noticed that constructing ASTD can avoid redundancies in HFPNe structure, while retaining expressiveness of dynamic behaviors.

In the next section, we demonstrate how to integrate and interpret the simulation data from circadian rhythm model in *Drosophila *to obtain a deeper understanding of structural and dynamic behaviors with ASTD.

## Results

### A case study: model of circadian clock

#### Biological background and modeling

**Figure 4:**
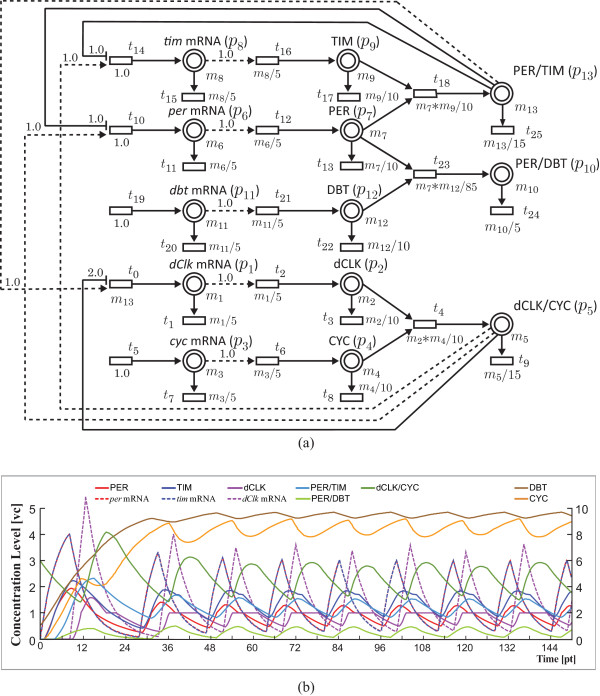
**HFPNe model and its simulation result of circadian rhythm in *Drosophila***. (a) HFPNe model of circadian rhythm in *Drosophila*. The accompanying variable *m*_*x *_at a place represents the concentration of the corresponding mRNA, protein or the compound. For example, the variable *m*_1 _indicates the concentration of *dClk *mRNA. Reaction speed (the rate of transcription, translation, complex formation or degradation) is expressed by a simple formula at each transition. For example, the formula *m*_1_/5 indicates the translation rate of dCLK protein that depends on the variable *m*_1 _for the *dClk *Mrna concentration. The real number over an arc is the threshold for the content of the place attached to this arc. For example, the translation of *tim *mRNA occurs during the period that the place value of *tim *mRNA exceeds 1.0. (b) Oscillations of *tim, per, dClk *mRNAs, and the proteins TIM, PER, dCLK, PER/TIM, PER/DBT, dCLK/CYC (left y-axis) and DBT, CYC (right y-axis). The unit of x-axis is [pt] ([pt] is the virtual time unit of the HFPNe model), while that of y-axis is [vc] ([vc] is the virtual concentration unit).

There are five genes involved in the *Drosophila *circadian rhythm: period (*per*), timeless (*tim*), *Drosophila *Clock (*dClk*), cycle (*cyc*) and double-time (*dbt*). It has been known that the *Drosophila *circadian system is composed of two interlocked negative feedback loops: (i) *Drosophila *proteins PER and TIM form a heterodimer (PER/TIM) in the cytoplasm. After the nuclear translocation, PER/TIM inhibits the transcription of *per *and *tim *in a cycling negative feedback loop. Meanwhile, PER/TIM activates the transcription of *dClk *involved in the dCLK/CYC negative feedback loop; (ii) the proteins dCLK and CYC form a heterodimer dCLK/CYC that activates *per *and *tim *transcriptions and inhibits *dClk *transcription. Figure 4(a) shows the HFPNe model of wild type (called *normal model*) without external disturbances (e.g., light effects). With parameters shown in Figure 4(a), *in silico *simulation generated stable oscillations in mRNAs of three clock genes, *tim, per, dClk*, and proteins dCLK, CYC, dCLK/CYC, PER, TIM, DBT, PER/DBT, PER/TIM with periods as shown in Figure 4(b). The detailed mechanism is given in [21-23].

#### Results and Discussion

##### [ASTD analysis of normal model]

**Figure 5:**
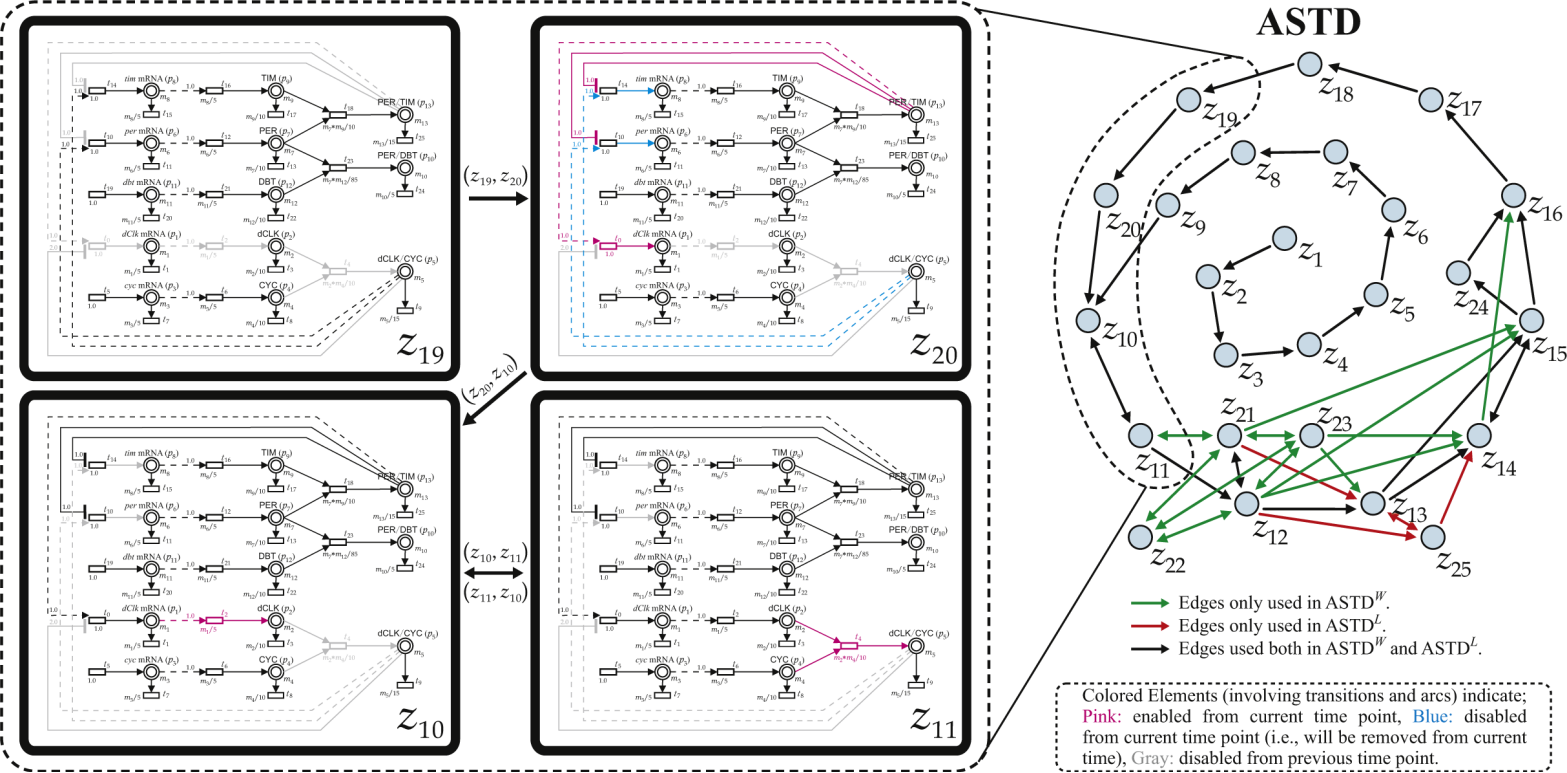
**Schematic representation of ASTD^*W*^and ASTD^*L*^**. The four bold-line blocks of *z*_19_, *z*_20_, *z*_10 _and *z*_11 _on the left side show the corresponding temporal subnets that are the minimal element sets at respective time points. Dashed-line block shows the state transitions of "*z*_19_→*z*_20_→*z*_10_↔*z*_11_" in the ASTD and corresponding detailed regulation variations of HFPNe elements. For example, in the structural transformation from *z*_19 _to *z*_20_, two inhibitory arcs *a*(*p*_13_, *t*_10_) and *a*(*p*_13_, *t*_14_) are enabled due to the increase in the concentration of PER/TIM, resulting in the deletion of four arcs *a*(*p*_5_, *t*_10_), *a*(*p*_5_, *t*_14_), *a'*(*t*_10_, *p*_6_) and *a'*(*t*_14_, *p*_8_). Note that the transformation from *z*_11 _to *z*_10 _is omitted.

This subsection presents the resulting ASTD from performing **Algorithm 1 **and **Algorithm 2**. The HFPNe model, simulation data and related data files of analyzing circadian rhythm model in *Drosophila *are available at the website [24]. In the simulation data, *x*_*sim *_is 150 [pt] with the time interval of 0.01 [pt] ([pt] is the virtual time unit of the HFPNe model). Figure 5 shows the resulting ASTD from processing the circadian time-course simulation data. The ASTD is derived by the following procedures: (i) By applying **Algorithm 1 **to the simulation data, we obtain the set of all the temporal subnets . The total number of the set is 15,000 corresponding to 15,000 time points. As mentioned above, each temporal subnet is the minimal element set at time *x*; and next (ii) Applying **Algorithm 2 **to the outputs of **Algorithm 1**, we construct the ASTD.

The constructed ASTD *G *= (*N*, *E*) of the normal model is composed of 24 unique nodes *N*={*z*_1_, *z*_2_,⋯, *z*_24_} connected by the black and green edges in *E *on the right side of Figure 5. Each node with a blue circle represents a state. The result demonstrates that our method successfully extracts 24 unique temporal subnets (i.e., ASNs) out of 15,000. This result suggests that (i) the number of all the temporal subnets of *Drosophila *circadian clock model can be reduced from 15,000 to only 24 when considering structural transformation due to the concentration variation, and further (ii) these 24 ASNs are all the states regulating the stable oscillations in the normal model. Each edge connected from previous state to current state denotes a direct structural transformation from the previous net structure to the current one. In Figure 5, dashed-line block is shown as an example to depict the state transitions of "*z*_19_→*z*_20_→*z*_10_↔*z*_11_" connected with the edges (*z*_19_, *z*_20_), (*z*_20_, *z*_10_), (*z*_10_, *z*_11_) and (*z*_11_, *z*_10_). Detailed direct structural transformations of HFPNe elements are illustrated in the left dashed-line block.

As an example we discuss the structural transformation from *z*_19 _to *z*_20_. Figures of the structural transformations of all the states along with corresponding ASTD are given as additional materials [see Additional files 2 and 3]. Due to an increase in PER/TIM concentration, two inhibitory arcs *a*(*p*_13_, *t*_10_) and *a*(*p*_13_, *t*_14_) are enabled (highlighted in pink), which results in the deletion of four arcs *a*(*p*_5_, *t*_10_), *a*(*p*_5_, *t*_14_), *a'*(*t*_10_, *p*_6_) and *a'*(*t*_14_, *p*_8_) (highlighted in blue). Meanwhile, a test arc from PER/TIM is also enabled, which results in the adding of arc *a*(*t*_0_, *p*_1_). It thus leads to a new temporal subnet structure regarded as *z*_20 _connected from the previous state *z*_19_. This captures the fact that in the *Drosophila *circadian clock model, the net structure in *z*_20 _can only transform from *z*_19 _on account of the rising of PER/TIM level. This restricts the reasonable transitions into state *z*_20 _to be *z*_19_, which simplifies analysis for both biologists and computational biologists.

The sparseness of this network will be of great value to computational biologists who need to rapidly investigating a range of regulatory interactions and dynamic behaviors from simulation data of their models. More detailedly, ASTD (i) gives researchers a concise impression of the connection relationship between the nodes. The nodes that researchers are interested in can be comprehensively focused and traced according to its connection in the ASTD; and (ii) such nodes can be further explored to explicitly elucidate the mechanism how the occurrence of structural transformation triggers oscillations along the time axis.

We also observe that state transitions from *z*_1 _to *z*_9 _are used only once, and are likely the period before the circadian rhythm systems reach a stable cycle from the initial marking. Excluding such nodes, the ASTD becomes only a cycle composed of the outer-ring nodes (i.e., all the nodes excluding {*z*_1_, *z*_2_,⋯, *z*_9_}). The state transfers following the cycle of these outer-ring nodes in the ASTD on the rhythms of mRNAs and proteins in this oscillation systems. In the following, we investigate how the ASTD changes when modifying the gene *dbt*.

##### [Mutant analysis]

Price *et al. *discussed the property of *dbt*^*L *^("L" for long) that is a mutation of *dbt*. They showed that the transcription of the gene *per *is affected by this mutant, i.e., the period of *per *mRNA in *dbt*^*L *^mutant is longer than the one in the normal model [23]. The behavior of *per *mRNA in *dbt*^*L *^mutant and normal model is validated afterwards by Matsuno *et al. *[22] (see Additional file 4 illustrating the simulation results). It is obtained by changing the formula at the transition (*t*_23 _= *m*_7_*m_12_/1000 in our case) denoting the complex forming rate of PER and DBT. Roughly speaking, when the forming rate of PER/DBT is slowed, it leaves more PER to bind to TIM, which leads to a faster increase of PER/TIM to a higher concentration. It thus will take longer time to inhibit the transcription of *per *mRNA and *tim *mRNA until the concentration of PER/TIM decreases to the respective threshold values of the inhibitor arcs (see Figure 4(a)). Therefore, the next increases of *per *mRNA and *tim *mRNA are accordingly postponed because of the longer inhibition effect resulting from the slow forming rate of PER/DBT. The results gave the suggestion that the circadian rhythm is controlled by this forming rate, which is affected by the mutant *dbt*^*L*^.

Due to the space limitation, we show the ASTD of *dbt*^*L *^mutant model (ASTD^*L *^for short) in the figure together with the ASTD of the normal model (denoted as ASTD^*W *^for short). Note that we employ the same mutant model in [22] for comparison. In Figure 5, the ASTD^*L*^(*N*, *E*) is composed of 23 distinct ASNs, where *N *= {*z*_1_, *z*_2_,⋯, *z*_21_, *z*_24_, *z*_25_} and *E *is the set of edges drawn in black and red. The resulting ASTD^*W *^and *ASTD*^*L *^give information that the temporal structure of ASTD^*L *^is simpler than the one of ASTD^*W *^from the viewpoints of node number and connection relationship. Figure 5 also provides the information to generate following views: (i) shared 22 nodes {*z*_1_, *z*_2_,⋯, *z*_21_, *z*_24_} in ASTD^*W *^and ASTD^*L*^contribute to produce oscillations regardless of the period length; and (ii) the temporal structures of disappeared *z*_22 _and *z*_23 _in ASTD^*L *^do not contribute to the regulation of the forming rate of complex PER/DBT. In Additional files 2 and 3, it can be confirmed that the transitions *t*_23 _in *z*_22 _and *z*_23 _are both disabled (in grey), which reflects that no complex forming action occurs by the formula alteration in the mutant model.

##### [Graphical-based analyses of ASTD]

As described above, ASTD can give the user concise impression regarding the time-dependent structural changes in the pathway, which provides a great help in investigating a range of regulatory interactions and dynamic system behaviors. Additionally, it would be helpful to consider some more intuitive graphical representations to express the characteristic information from ASTD. With the help of such characteristics, one can obtain an intuitive understanding to the dynamic behaviors such as "Which state is maintained longer?", "How does the frequency of entering a certain state vary with the parameters?", and "How does the concentration of a substance vary in each state?"

We incorporate graphical representation applying to the states in the ASTD showing three characteristics: (i) duration, (ii) out-degree, and (iii) total concentration difference of substance in each state.

**Figure 6:**
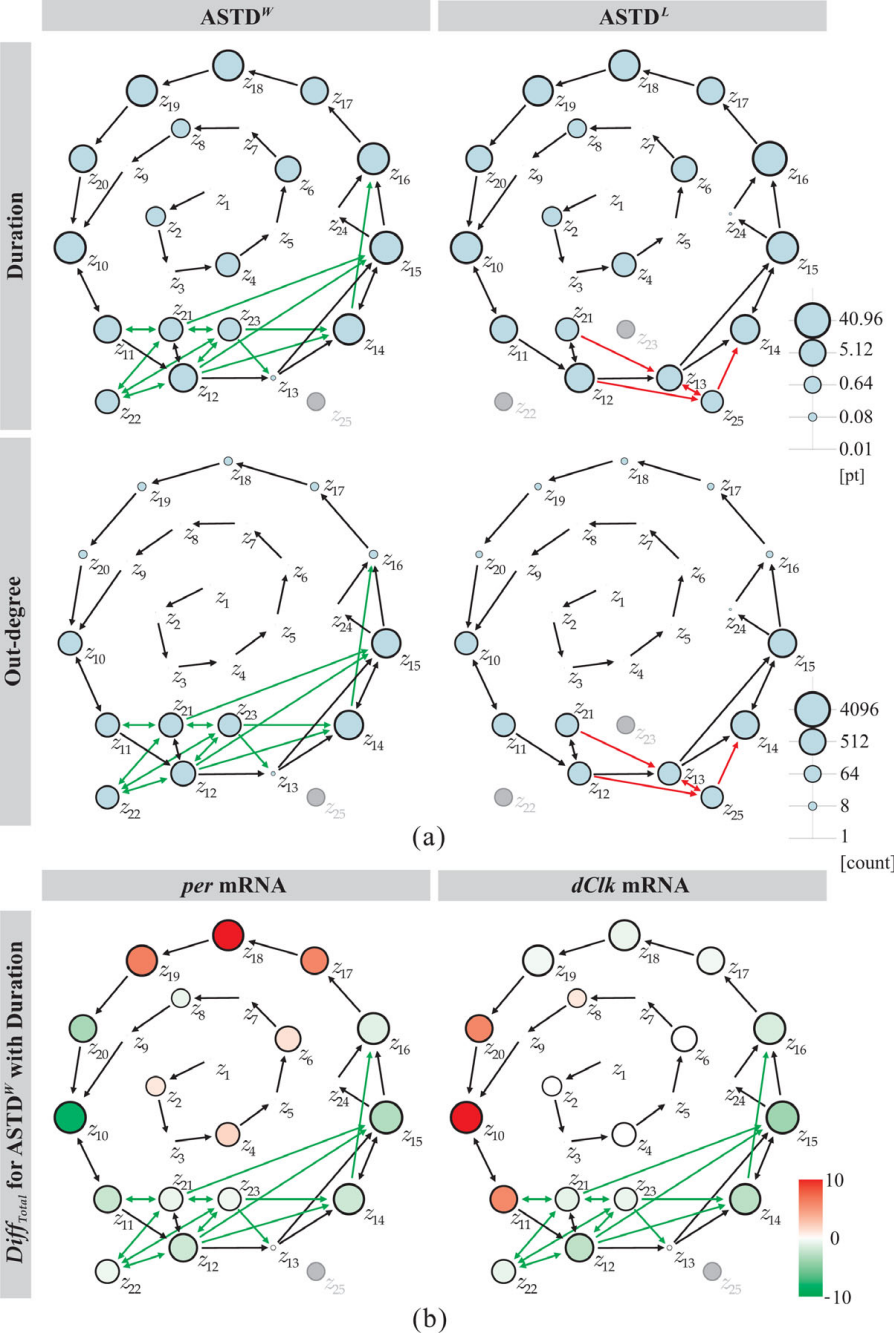
**Three characteristic overviews of the ASTD for the circadian rhythm model**. (a) ASTD^*W *^and ASTD^*L *^characterized with respect to duration (upper) and out-degree (lower). (b) ASTD^*W *^characterized with duration and the total concentration difference for *per *and *dClk *mRNAs.

Firstly, *duration *is used to demonstrate the total persistence period of each state when it is reached. Node size of ASTD is then scaled up or down corresponding to the duration summation of each state. Secondly, *out-degree *is the number of edges going out of a node. This concept is employed to characterize the frequency of a certain node used in ASTD. Figure 6(a) displays the characterized ASTD^*W *^and ASTD^*L *^with respect to the duration and out-degree. The scales of node sizes denoting duration and out-degree are given on the right-side of Figure 6(a), respectively. The node size of duration is according to the persistence time period of each node. The node size of out-degree is based on the calculation of natural log *ln*(*count*), where *count *is the number of edges going out of a node. We demonstrate how to analyze ASTD in general by using duration together with out-degree, as well as discussing obtained ASTD^*W *^and ASTD^*L *^of circadian rhythm in *Drosophila *shown in Figure 6(a) as follows:

(i) When duration is "B" (B for the big size of the node) and the out-degree is "S" (S for the small size of the node), the state of such node (e.g., *z*_18 _and *z*_19_,) is maintained at a relative stable condition;

(ii) When duration and out-degree are both "S", the state of such node (e.g., *z*_3 _and *z*_13_) is seldom-used during the simulation and is negligible. *z*_3 _with both "S" duration and out-degree in ASTD^*W *^and ASTD^*L*^, is considered negligible. On the other hand, *z*_13 _displays "B" duration and out-degree in ASTD^*L *^unlike in ASTD^*W*^. As shown in Additional file 4, *per *mRNA has a longer periodic oscillation in the mutant model than in the normal one. In ASTD^*L*^, the state *z*_15 _is transformed from *z*_13 _and *z*_14_. Not only duration but also out-degree of *z*_14 _in ASTD^*W *^and ASTD^*L *^are almost in the same size. But those of *z*_13 _changed from "S" in the normal model to "L" in the mutant one, and the node size is closed to *z*_14_. It can be considered that the temporal structure of *z*_13 _contributes to the regulatory mechanism to generate longer periodic oscillations of *per *mRNA in the mutant model;

(iii) The case of that duration is "S" and out-degree is "B" is not observed in this ASTD. Such nodes are highly unstable and trigger frequent structural transformations. This instability is likely the reason such nodes are not found in the stable oscillations of the circadian rhythm;

(iv) When duration and out-degree are both "B", the state of such node (e.g., *z*_12 _and *z*_14_) is unstable, but it is likely such node is important to the regulation of the oscillations.

Note that the ASTD allows multiple edges between two nodes. Several bidirectional arcs are observed in the resulting ASTD, e.g., the arcs between *z*_14 _and *z*_15_, *z*_21 _and *z*_23_, which represent two states oscillates from one another. We examined there occurs a series of short-term tiny concentration vibrations of PER and dCLK around respective threshold values, which lead to the bidirectional arcs coming into being. For example, when the concentration of dCLK (*p*_2_) changes up and down around evaluated threshold value 1.0 of the arc (*p*_2_, *t*_4_), the states *z*_14 _and *z*_15 _will oscillates from one another. The results of out-degree in Figure 6(a) shows that the nodes connected with bidirectional arcs are relatively larger than others. These larger nodes reflecting unstable short-term tiny concentration vibrations are considered to contribute to the formation of the steady oscillation period (i.e., the cycle composed of the outer-ring nodes).

Finally, we investigate the dynamic behaviors of each substance's concentration level. We present this in a heat-map-like representation, where the *total concentration difference *is represented by colors as shown in Figure 6(b). The total difference in concentration level *Diff*_*Total*_(*p*, *z*) is the difference summation in place *p'*s concentration (*M*(*p*) [*x*_*i*_]-*M *(*p*) [*x*_*i*-1_]) at adjacent time points in the given state *z*. Figure 6(b) compares concentration differences in *per *mRNA (*p*_1_) and *dClk *mRNA (*p*_6_). In tracking *per *mRNA, the nodes *z*_17_, *z*_18 _and *z*_19 _are colored in red around *z*_18_, while for *dClk *mRNA, the nodes *z*_20_, *z*_10 _and *z*_11 _are colored in red around *z*_11_. The shift states accurately reflect the difference in rise times for *per *and *dClk *mRNA level. Further, by investigating the temporal subnets in the ASTD, it is confirmed that the transition *t*_10 _denoting the transcription of *per *mRNA is enabled only in the red states *z*_17_, *z*_18 _and *z*_19_, while *t*_10 _is disabled in all the other states due to the inhibition of PER/TIM. Similarly, the transition *t*_0 _of *dClk *mRNA transcription is enabled in the red states *z*_20_, *z*_10 _and *z*_11_, while it is disabled in the others because of the inhibition by the dCLK/CYC complex. In this way, the information of each substance's relative expression can be easily visualized using ASTD with this characteristic.

## Discussion

Investigating dynamic behaviors of biological networks is usually achieved by analyzing concentration plots of the simulation data. However, studying such concentration variations of a model generate more vital temporal structural information than considering dynamics as an ensemble. We thus proposed a novel techniques combines quantitative simulation data and topological analysis to deduce the dynamic behaviors of system mechanisms from the data. In this paper, we give a cycle ASTD of circadian rhythm of *Drosophila *from the simulation data as an example. ASTD can also be a linear succession of states, which is usually derived from the signaling pathway owing to its feature of propagating signals from transmembrane to the DNA nucleus. Such linear ASTD can also be employed as an analysis tool for quick interpretation. With the aid of graphical-based analyses, ASTD can yield concise impression of the connection relationship among the states from various viewpoints, such as time period, concentration variation and so on.

### ASTD and reachability graph

ASTD is different from the concept of reachability graph (i.e., graph of markings) [25]. ASTD is made up of the nodes that are the unique temporal subnets, and of directed edges corresponding to the structural transformation of temporal subnets resulting in the passing from one state to another. Each node in ASTD is the grouping of identical temporal subnets from the viewpoint of structure. That is, each node simply possesses the structural information of the entries in the minimal element set that is extracted by eliminating the disabled transitions/arcs and isolated places. No concentration information (i.e., marking) is recorded in the ASTD (this is the point different from the concept of reachability graph) although such information is used to determine the ASTD. In contrast, reachability graph consists of nodes corresponding to reachable markings and of arcs corresponding to firing of transitions [25]. From this case study with the given sampling interval, the state space of reachability graph is 15,000, while that of ASTD^*L *^is significantly reduced to 24. A series of non-negative real numbers in the column of the EDF file is equivalent to current marking (state) in the reachability graph. Additionally, ASTD can deal with any general type, e.g., string and object. There are totally 15,000 unique markings obtained from EDF file. The state space of reachability graph will not be less than 15,000 when further decreasing the sampling interval time of simulation.

## Conclusions

This paper describe a novel methodology to construct a so-called *active state transition diagram *(ASTD) by using the time-course simulation data from a well-founded formal framework of hybrid functional Petri net with extension (HFPNe). The main contributions are as follows: (i) Automatically constructed ASTD we have presented suggests that building an ASTD representation can eliminate redundant HFPNe structures, while maintaining equivalent expressiveness as the full model; (ii) Characterized ASTD gives the user concise impression and new insights to grasp and trace how a key regulatory subnet and/or a network changes with time; (iii) Due to the nature of the ASTD, any state belonging to the ASTD is able to be simulated and it enables us to simulate equivalent concentration distributions; and (iv) The applicability of the proposed method is investigated by the analysis of an HFPNe model of circadian rhythm in *Drosophila*.

Another approach to represent biochemical reactions as a system is to use a series of ordinary differential equations (ODEs). Since the HFPNe allows quantities to be continuous and generic, the biological processes with ODE-based kinetics can be realized [22], i.e., an ODE-based system that is convertible into an HFPNe model, can yield an ASTD and give simplified graphical representation of the time-dependent structural transformation. There is, however, a special case at this point, for models without inhibitory arcs and with threshold value of normal and test arcs equal to zero, the resulting ASTDs will contain only one state - a full HFPNe structure - for all the time points. Moreover, for the ODE model of hybrid dynamical systems, an existing method has been developed providing a mathematical approach with applying reachability analysis by Halász *et al. *[16]. It serves as a promising theoretic basis and leads us to make further investigation on this special case as the future work.

In this paper, the circadian rhythm model of *Drosophila *is a deterministic one, in which all the parameters of transition speeds and arc thresholds have been determined in advance [22]. Since HFPNe model supports stochastic transitions as well, in the future, ASTD will be adapted to such probabilistic features of the system as well as the firing conflict problem of the discrete transition by means of particular graphical-based representation. Additionally, building ASTD makes possible converting a hybrid model dealing with discrete, continuous and more complicated events to finite time-dependent states. Various analysis techniques, e.g., network motif analysis, centrality analysis, clustering analysis and model checking technique, will also be imported to the ASTD to obtain better understanding of systematic dynamics from simulation data as the future research.

## Authors' contributions

The basic idea was considered by MN and further developed by CL and MN. MN implemented proposed method, and CL wrote the draft of the manuscript. The most of the figures are made by AS. CL, MN and AS evaluated the resulting ASTD. SM supervised the whole study. The final manuscript was read and approved by all authors.

## Supplementary Material

Additional file 1 **Definition of hybrid functional Petri net with extension (HFPNe)**. The data provide full mathematical definitions of HFPNe. Click here for file

Additional file 2 **Detailed net structure of the nodes in the resulting ASTD**. The detailed net structure of nodes (i.e., *z*_10_,⋯, *z*_25_) in the resulting ASTD shown in Figure 5. The connection relationships are shown between the bold-line blocks of the nodes. Legend is given in Figure 5. Click here for file

Additional file 3 **Detailed net structure of all the nodes in the resulting ASTD**. Detailed net structure of all 25 nodes (note that one node is displayed in one page). Readers can turn page forward and back to see the structural difference between two nodes in an easy-to-understand manner. Click here for file

Additional file 4 **Concentration behaviors of *per *mRNA: (a) normal model; and (b) *dbt*^*L *^mutant**. Formula such as *m*_7 _* m_12_/1000 for the firing speed of transition *t*_23 _is given at charts (a) and (b), which represents complex forming rate of two proteins PER and DBT. The firing speed in *dbt*^*L *^is slower than the one in the normal model. Click here for file
