# Supplementary material for: Time-dependent structural transformation analysis to high-level Petri net model with active state transition diagram
Source: BMC Syst Biol. 2010 Apr 1;4:39. doi: 10.1186/1752-0509-4-39 (PMC2855528; doi:10.1186/1752-0509-4-39)
Supplement: Additional file 1 — Definition of hybrid functional Petri net with extension (HFPNe). The data provide full mathematical definitions of HFPNe. [file 1752-0509-4-39-S1.PDF]

Additional File 1

1 Definition of HFPNe

The definition of HFPNe mainly consists of two parts. The first is the definition of *types* that can be used as data types in HFPNe. Using the defined types, we can handle various biological entities. The second is to use the function f to determine the parameters such as weight, delay, and speed, which control the system behavior. Using the defined function f, we can handle complicated biological events.

We first define a terminology with which we define the notion of HFPNe formally.

[Definition I] The set $\mathcal{T}$ of *types* is defined as follows:

$\langle type \rangle$ ::= `boolean`$\|$ `integer`$\|$ `integer+`$\|$ `real`$\|$ `real+`$\|$ `string`$\|$ `pair`$(\langle$`type`$\rangle, \langle$`type`$\rangle)\|$ `list`$\langle$`type`$\rangle\|$ `object`$(\langle$`type`$\rangle, \cdots, \langle$`type`$\rangle)$. □

[Definition II] For each $\theta \in \mathcal{T}$, we associate it with its *domain* $D(\theta)$ as follows:

1. $D(\texttt{boolean}) = \{\texttt{true}, \texttt{false}\}$.
2. $D(\texttt{integer}) = \mathbf{Z}$ (the set of integers).
3. $D(\texttt{integer+}) = \mathbf{N}$ (the set of nonnegative integers).
4. $D(\texttt{real}) = \mathbf{R}$ (the set of real numbers).
5. $D(\texttt{real+}) = \mathbf{R}^{\geq 0}$ (the set of nonnegative real numbers).
6. $D(\texttt{string}) = \mathbf{S}$ (the set of strings over some alphabet).
7. $D(\texttt{pair}(\theta_1, \theta_2)) = D(\theta_1) \times D(\theta_2)$.
8. $D(\texttt{list}\theta) = \bigcup_{k \geq 0} D(\theta)^k$.
9. $D(\texttt{object}(\theta_1, \cdots, \theta_n)) = D(\theta_1) \times \cdots \times D(\theta_n)$.

We denote $D^* = \bigcup_{\theta \in \mathcal{T}} D(\theta)$. □

For HFPNe, we newly introduce places and transitions which deal with D^* and these places and transitions are named "generic" while the names "discrete" and "continuous" are used for places and transitions of HFPN (hybrid functional Petri net).

Let P be a finite set of places. Each place is labeled with either `discrete`, `continuous`, or `generic`.

Place labeled with `discrete` (resp., `continuous`, `generic`) is called *discrete* (resp., *continuous*, *generic*). We assign a type $\tau(p)$ to each place p in P by a mapping called a *type function* $\tau : P \to \mathcal{T}$ which satisfies the following conditions:

1. If p is labeled with `discrete`, then $\tau(p) = \texttt{integer+}$.
2. If p is labeled with `continuous`, then $\tau(p) = \texttt{real+}$.
3. If p is labeled with `generic`, it can be any type in $\mathcal{T}$.

A *marking* of P is defined as a mapping $M : P \to D^*$ such that $M[p]$ is in $D(\tau(p))$ for $p \in P$. $M[p]$ is called the *mark* of p. We denote the set of all markings of P by $\mathcal{M}$.

In advance, we need define some notations which will be used for the definition of HFPNe. Let $\mathcal{D}_{\texttt{discrete}} = \{f \mid f : \mathcal{M} \to \mathbf{N}\}$, $\mathcal{D}_{\texttt{boolean}} = \{b \mid f : \mathcal{M} \to \{\texttt{true}, \texttt{false}\}$, and $\mathcal{D}_{\texttt{generic}} = \{f \mid f : \mathcal{M} \to D^*\}$.

Consider $f : \prod_{p \in P} D(\tau(p)) \to \mathbf{R}^{\geq 0}$. Note that the set $\mathcal{M}$ can be identified with the set $\prod_{p \in P} D(\tau(p))$. Let R be the set of all places labeled with `continuous` and let $Q = P - R$. For an element $v \in \prod_{p \in Q} D(\tau(p))$, let $f[Q = v] : \prod_{p \in R} D(\tau(p)) \to \mathbf{R}^{\geq 0}$ be the function defined by $f[Q = v](v') = f(v', v)$ for $v' \in \prod_{p \in R} D(\tau(p))$. We say that f is *continuous* if $f[Q = v]$ is continuous on $\prod_{p \in R} D(\tau(p))$ for any $v \in \prod_{p \in Q} D(\tau(p))$. Then let $\mathcal{D}_{\texttt{continuous}} = \{f \mid f : \mathcal{M} \to \mathbf{R}^{\geq 0}$ is continuous$\}$.

Based on the above notions, we define HFPNe by introducing data types for places by giving functions which depend on markings to determine the weight, delay, and rate, etc. The definition of HFPNe is as follows:

[Definition III] A *hybrid functional Petri net with extension* (HFPNe)

$$H = (P, T, A, \tau, w, u, d)$$

consists of the following:

1. P is a finite set of *places* and T is a finite set of *transitions*. We assume $P \cap T = \emptyset$. Each place is labeled with either `discrete`, `continuous`, or `generic`. Each transition is also labeled with `discrete`, `continuous`, or `generic`. The transition and place are called *discrete*, *continuous*, or *generic* according to its label.

   For each transition t in T, it has two sets $Input_t$ and $Output_t$ of arcs. Arc $a \in Input_t$ is an edge from input place p_a to the transition t called *input arc*. Arc $a' \in Output_t$ is an edge from the transition t to output place $p_{a'}$ called *output arc*. Each arc is labeled with either `normal`, `test`, or `inhibitory`, and arc labeled with `normal` (resp., `test`, `inhibitory`) is called *normal arc* (resp., *test arc*, *inhibitory arc*). We also say that arcs (a and a') are *discrete* (resp., *continuous*, *generic*) if transition t is discrete (resp., continuous, generic). For graphical representation, we use the symbols in Figure 1. The labels of arcs, places and transitions satisfy the following rules:

   a. The label of arc $a' \in Output_t$ is `normal`.

   b. For arc $a \in Input_t$ with input place p_a, the labels of arc a, place p_a, and transition t satisfies the connection rules in Table 1(a).

   c. For arc $a' \in Output_t$ with output place $p_{a'}$, the labels of arc a', place $p_{a'}$ and transition t satisfies the connection rules in Table 1(b).

   We denote by PT and TP the set of input arcs and the set of output arcs of all transitions, respectively. We also denote arc a in PT as $a(p, t)$ by specifying input place and transition. In a similar way, arc a' in TP is denoted as $a'(t, p)$ by specifying transition and output place. The set A of arcs is given by $PT \cup TP$.

2. The types of places are given by a type function $\tau : P \to \mathcal{T}$.

3. For each input arc $a \in PT$, its *activity* $w(a)$ is given by an *activity function* $w : PT \to \mathcal{D}_{\texttt{discrete}} \cup \mathcal{D}_{\texttt{continuous}} \cup \mathcal{D}_{\texttt{boolean}}$ which satisfies the following conditions:

   a. If a is discrete, $w(a)$ is in $\mathcal{D}_{\texttt{discrete}}$.

   b. If a is continuous, $w(a)$ is in $\mathcal{D}_{\texttt{continuous}}$.

   c. If a is generic, $w(a)$ is in $\mathcal{D}_{\texttt{boolean}}$.

Table 1. "$\surd$" and "$-$" show that the connections are allowed and disallowed, respectively. For example, the left top "$\surd$"s in (a) and (b) mean that a discrete place can connect to a discrete transition via a normal arc and a test or inhibitory arc, respectively. The left top "$\surd$" in (c) means that a normal arc from a discrete arc can connect to a discrete place. The left bottom "$-$" in (a) means that a generic place cannot connect to a discrete transition via a normal arc.

(a) Normal input arc $p_a \xrightarrow{a} t$.

label of arc a		normal		
label of transition t		discrete	continuous	generic
label of place p_a	discrete	$\surd$	$-$	$\surd$
	continuous	$\surd$	$\surd$	$\surd$
	generic	$-$	$-$	$\surd$

(b) Test or inhibitory input arc $p_a \xrightarrow{a} t$.

label of arc a		test or inhibitory		
label transition t		discrete	continuous	generic
label of place p_a	discrete	$\surd$	$\surd$	$\surd$
	continuous	$\surd$	$\surd$	$\surd$
	generic	$\surd$	$\surd$	$\surd$

(c) Output arc $t \xrightarrow{a'} p_{a'}$.

label of arc a'		normal		
label of transition t		discrete	continuous	generic
label of place $p_{a'}$	discrete	$\surd$	$-$	$\surd$
	continuous	$\surd$	$\surd$	$\surd$
	generic	$-$	$-$	$\surd$

For input arc $a(p,t)$, $w(a)$ is used as a function giving the threshold in discrete and continuous cases and the condition in generic case which is required for enabling the transition t.

4. For each arc c ($c = a(p,t) \in PT$ or $c = a'(t,p) \in TP$), the *update* $u(c)$ is given by an *update function* $u : A \to \mathcal{D}_{\texttt{discrete}} \cup \mathcal{D}_{\texttt{continuous}} \cup \mathcal{D}_{\texttt{generic}}$ which satisfies the following conditions:

   a. If c is discrete, $u(c)$ is in $\mathcal{D}_{\texttt{discrete}}$.
   b. If c is continuous, $u(c)$ is in $\mathcal{D}_{\texttt{continuous}}$.
   c. If c is generic, then $u(c)$ is in $\mathcal{D}_{\texttt{generic}}$ such that $u(c)[M]$ is in $D(\tau(p))$ for any marking $M \in \mathcal{M}$. $u(c)$ is used as a function which will update the mark of p.

5. For each discrete or generic transition t, the *delay* of t is given by a *delay function* $d : T_{\texttt{discrete}} \cup T_{\texttt{generic}} \to \mathcal{D}_{\texttt{continuous}}$, where $T_{\texttt{discrete}}$ (resp., $T_{\texttt{generic}}$) is the set of discrete transitions resp., generic transitions). The delay of firing on marking M is given by $d(t)[M]$.

We use the parameter $x \geq 0$ for the *time* in HFPNe. Do not confuse t for transition with x for time.

A *marking* of P is defined as a mapping M that assigns a mark (the type of contents) to each place. $M[p]$ is called the *mark* of p. The *initial marking* I is a marking at time $x = 0$ and we denote the *marking at time* x by $M(x)$. The *reserved marking* $M_r(x)$ at time x represents the amount of "tokens" reserved for firing when firing conditions are satisfied. By convention, let $M(p,x)$ be $M[p](x)$, and $M_r(p,x)$ be $M_r[p](x)$ for $p \in P$. We define $\tilde{M}(x)$ by $\tilde{M}[p](x) = M[p](x) - M_r[p](x)$ if p is discrete or continuous and $\tilde{M}[p](x) = M[p](x)$ if p is generic. Given the initial marking of HFPNe, the marking $M(x)$ and the reserved marking $M_r(x)$ at time x are defined in the following way:

For time $x = 0$, $M(0) = I$ by definition. We define $M_r[p](0) = 0$ if p is discrete or continuous, and $M_r[p](0) = \texttt{null}$ (the empty list) if p is generic. For $x>0$, we define $M(x)$ and $M_r(x)$ in the following way. For transition t at time x, we say that t is *enabled at time* x if the following conditions are satisfied. Otherwise the transition is said to be *disabled at time* x.

1. If t is discrete or continuous, then for all input arcs $c = a(p,t) \in PT$ the following conditions hold:

   a. $\tilde{M}[p](x) > w(c)[M(x)]$ if a is not labeled with `inhibitory`;
   b. $\tilde{M}[p](x) < w(c)[M(x)]$ if a is labeled with `inhibitory`, where $w(c)[M(x)]$ is the threshold value of c on marking M at time x.

2. If t is generic, then for all input arcs $a(p,t) \in PT$ the following conditions hold:

   a. $w(a)[\tilde{M}(x)] = \texttt{true}$ if a is not labeled with `inhibitory`;
   b. $w(a)[\tilde{M}(x)] = \texttt{false}$ if a is labeled with `inhibitory`.

$\square$

[Definition IV] If disabled transition t turns enabled at time x, we say that t is *triggered at time* x and x is called the *trigger time*. If enabled transition t turns disabled at time x, we say that t is *switched off at time* x and x is called the *switch-off time*. $\square$

[Definition V] We define *firing* of discrete transition t. Assume that discrete transition t is triggered at time x. For each normal input arc $a(p,t)$, the place p must be discrete or continuous by definition. Then $M_r[p]$ reserves $\alpha \cdot u(a)[M(x)]$, i.e. $\alpha \cdot u(a)[M(x)]$ is added to $M_r[p]$, for the time $y > x$ until $x + d(t)[M(x)]$, where $\alpha = \{0, 1\}$, if $\alpha = 0$, reserve is disabled; otherwise, token is reserved. If t is enabled at $x + d(t)[M(x)]$, then at the same time $x + d(t)[M(x)]$, $M[p]$ is decreased by $u(a)[M(x)]$ and $M_r[p]$ releases $u(a)[M(x)]$, i.e., $u(a)[M(x)]$ is decreased from $M_r[p]$. Simultaneously, for each output normal arc $a'(t,p')$, $M[p']$ is increased by $u(a')[M(x)]$ at time $x + d(t)[M(x)]$ by arc $a'(t,p')$. The time $d(t)[M(x)]$ is called the *delay* that is determined by the function $d(t)$ of the mark $M(x)$ at time x.

As we will describe in Definitions VII and VIII below, the reservation is not performed by generic or continuous transition. However, for the place p, there may be another discrete transitions $t_1, \ldots, t_\ell$ with normal input arcs $a_1(p,t_1), \ldots, a_m(p,t_\ell)$ which are triggered at time x. Then each discrete transition t_i tries to reserve $u(a_i)[M(x)]$ from the same $M[p]$ at time x for $i = 0, \ldots, \ell$, where $a_0 = a(p,t)$ and $t_0 = t$. We say that there is a *conflict* with p at time x if $M[p](x) < \sum_{k=0}^{\ell} u(a_i)[M(x)]$. When a conflict occurs, some *conflict resolution* should be applied, e.g., random selection of transitions, priorities on transitions, etc.

Even if some conflict resolution procedure selected the transition t to go further, the place p of $a(p,t)$ may be input places or output places of another discrete/continuous/generic transitions. By this, $M[p]$ and $M_r[p]$, and therefore $\tilde{M}[p]$, may be changed, the conditions of "enabled" are not be necessarily satisfied until

the firing time $x + d(t)[M(x)]$. When t becomes disabled before $x + d(t)[M(x)]$, we say that a *system error* occurs with t.

Thus triggered transition does not necessarily fire. If all of these actions succeed, we say that t *fires* at time $x + d(t)[M(x)]$. □

[Definition VI] We define *firing* of generic transition t. Assume that generic transition t is triggered at time x. For each normal input arc $a(p, t)$, the place p can be discrete, continuous and generic. For each output normal arc $a'(t, p')$, p' can be also any kind of places. If t keeps enabled until time $x + d(t)[M(x)]$, then $M[p]$ at time $x + d(t)[M(x)]$ is updated to $u(a)[M(x)]$ and $M[p']$ is updated to $u(a')[M(x)]$ at time $x + d(t)[M(x)]$. We say that t *fires* at time $x + d(t)[M(x)]$ if this action succeeds. If p is generic, it is always that $M_r[p](x) = \texttt{null}$. No change is added to $M_r[p]$ by arc $a(p, t)$ if p is discrete or continuous.

In a similar way to discrete transition, if p is discrete or continuous, Mp and $M_r[p]$ have a possibility to be changed before $x + d(t)[M(x)]$ by another transitions. Therefore $w(a)[\tilde{M}(y)] = \texttt{true}$ is not necessarily kept for $y \in (x, x + d(t)[M(x)])$. As in the case of discrete transition, it should be reported as system error. Since generic transition updates $M[p]$ and $M[p']$ at time $x + d(t)[M(x)]$, there is a possibility of conflict with another transitions which use p and p'. Thus some conflict resolution should be applied or it should be reported as system error. □

[Definition VII] We define *firing* of continuous transition t. When continuous transition t is triggered, it starts firing and updates the marks of its connected places continuously with the speeds determined by the update function u and the marking M as long as it is enabled. Assume that continuous transition t is enabled at time x. For each normal input arc $a(p, t)$, the place p must be continuous by definition. Then the mark $M[p]$ will be decreased through the arc $a(p, t)$ with the additional speed $u(a)[M(x)]$ at time x. No change is added to $M_r[p]$ by arc $a(p, t)$. For output normal arc $a'(t, p')$, the place p' must be continuous by definition. Then the mark $M[p']$ will be increased through the arc $a'(t, p')$ with the additional speed $u(a')[M(x)]$ at time x. No change is added to $M_r[p']$ by arc $a'(t, p')$.

As we have investigated in Definitions VI and VII, we consider continuous place p at time x. Continuous place p may be connected to some transitions with normal input/output arcs. Then let $t_1, \ldots, t_i$ be the continuous transitions which are enabled at time x and have p as input place with normal input arcs $a_1(p, t_1), \ldots, a_i(p, t_i)$. Since continuous transition has no delay in firing, we consider continuous transitions $t'_1, \ldots, t'_j$ which have p as output place with output normal arcs $a'(t'_1, p), \ldots, a'(t'_j, p)$ and are enabled at time x. Then the decreases and increases through these normal input and output arcs are summed up to define the derivative of $M[p]$ at time x:

$$\frac{dM[p](x)}{dx} = -\sum_{k=1}^{i} u(a_k)[M(x)] + \sum_{k=1}^{j} u(a'_k)[M(x)].$$

Starting from the time $x = 0$, we can solve the above equation (numerically) by using the initial mark $M(0)$ as the initial condition until one of the following event occurs if no system error occurs:

E1: A new continuous transition is triggered or some already enabled continuous transition is switched off. This may change the definition of the equation.

E2: Some discrete or generic transition is triggered or fires. This may change the initial condition for solving the equation.

Thus we can take a series of time points $x_0, x_1, \cdots$ such that E1 and/or E2 occur at time x_i and neither E1 nor E2 occurs in the time interval (x_i, x_{i+1}). Then we can define the equation for the time interval (left closed and right open) $[x_i, x_{i+1})$ and solve it with $M(x_i)$ as the initial condition. □
