# Supplementary figures and images for: Time-dependent structural transformation analysis to high-level Petri net model with active state transition diagram
Source: BMC Syst Biol. 2010 Apr 1;4:39. doi: 10.1186/1752-0509-4-39 (PMC2855528; doi:10.1186/1752-0509-4-39)

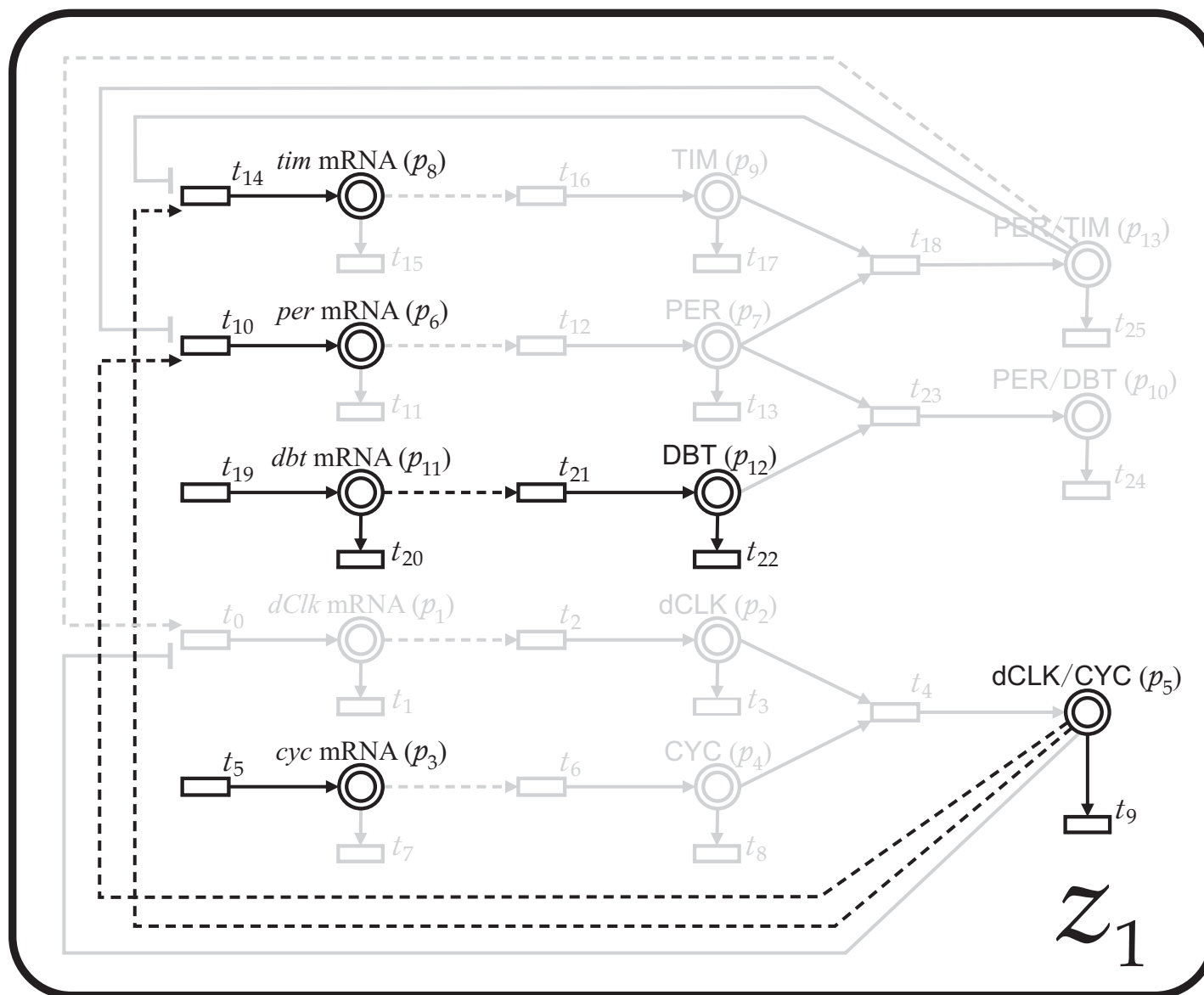

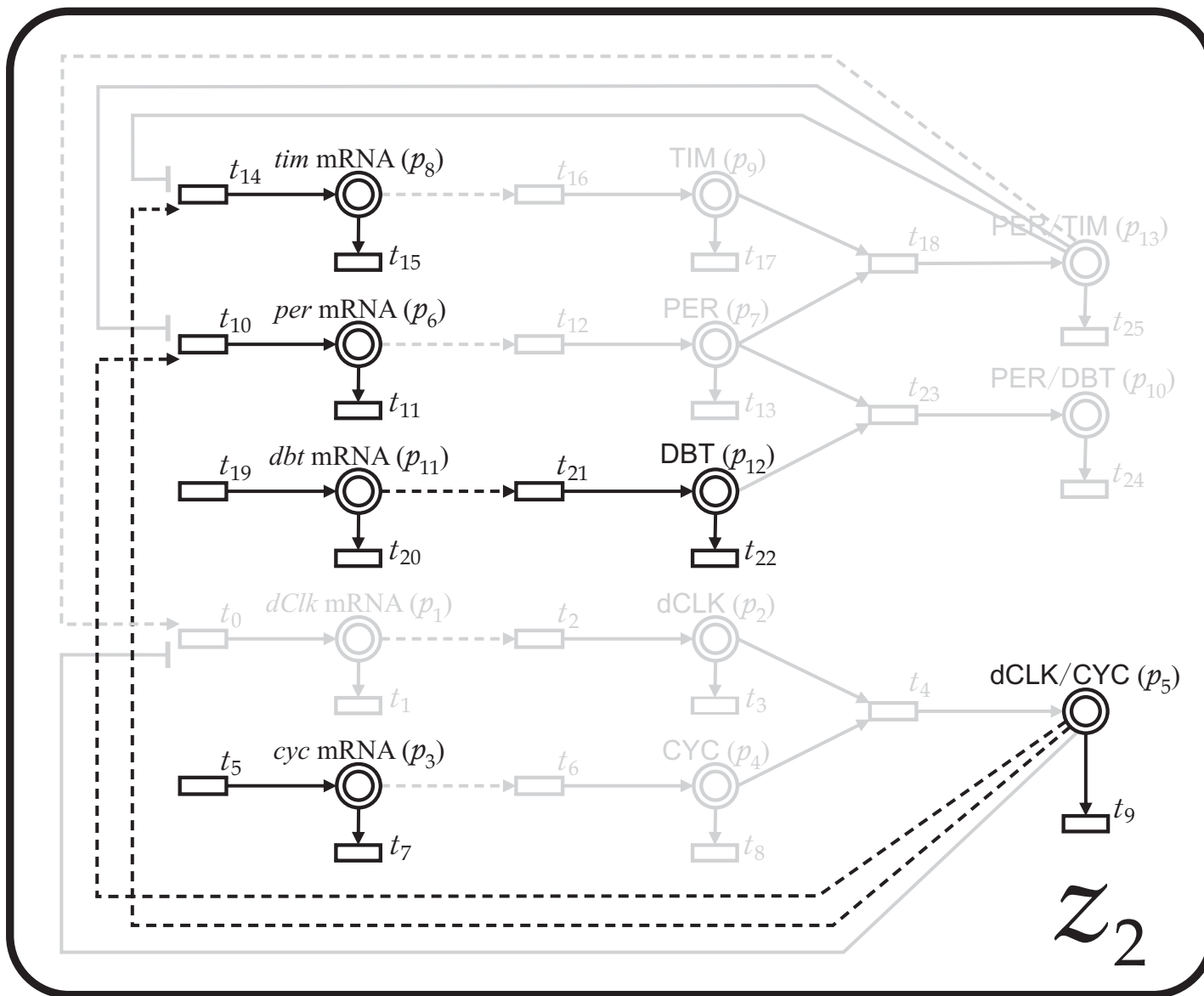

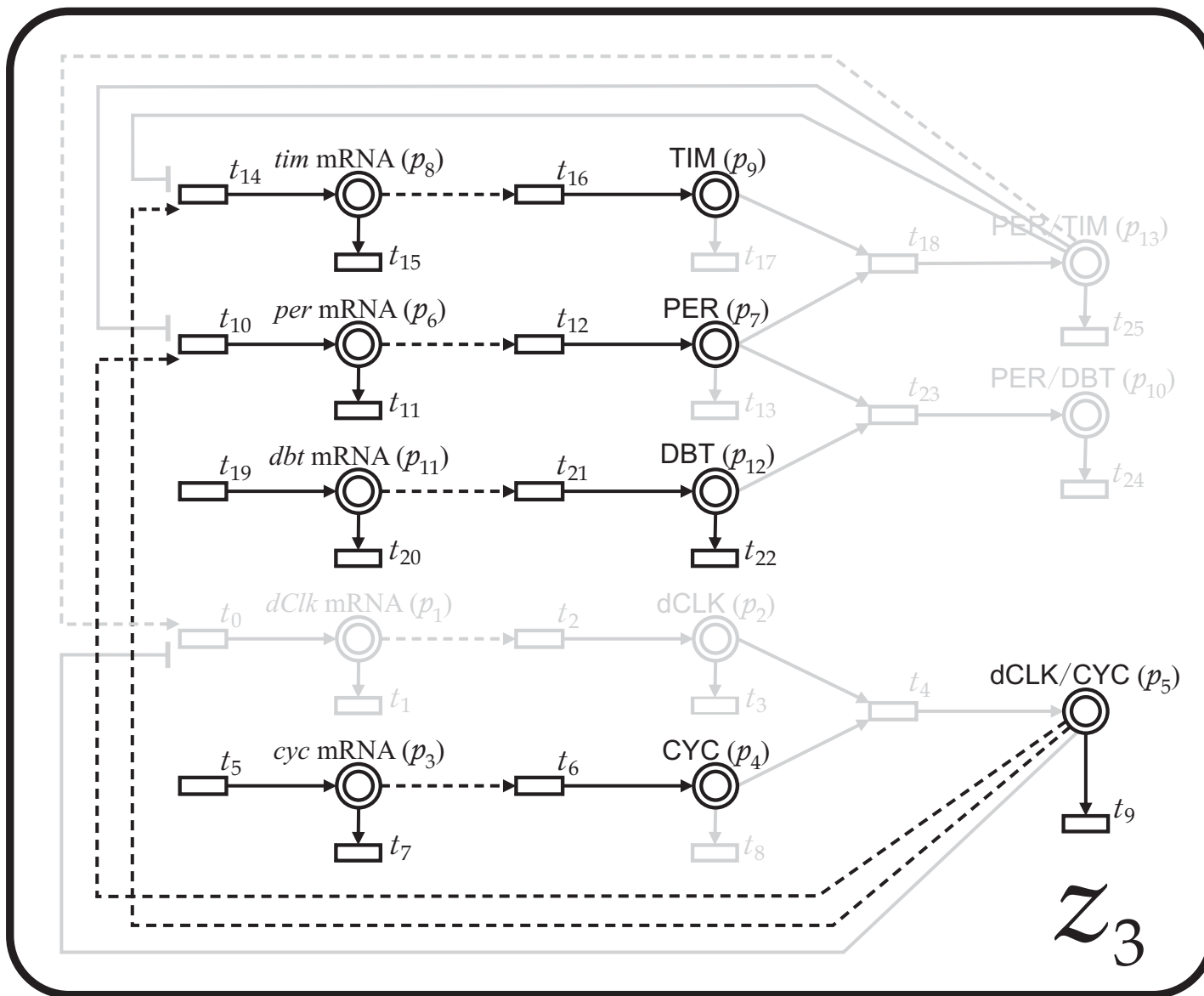

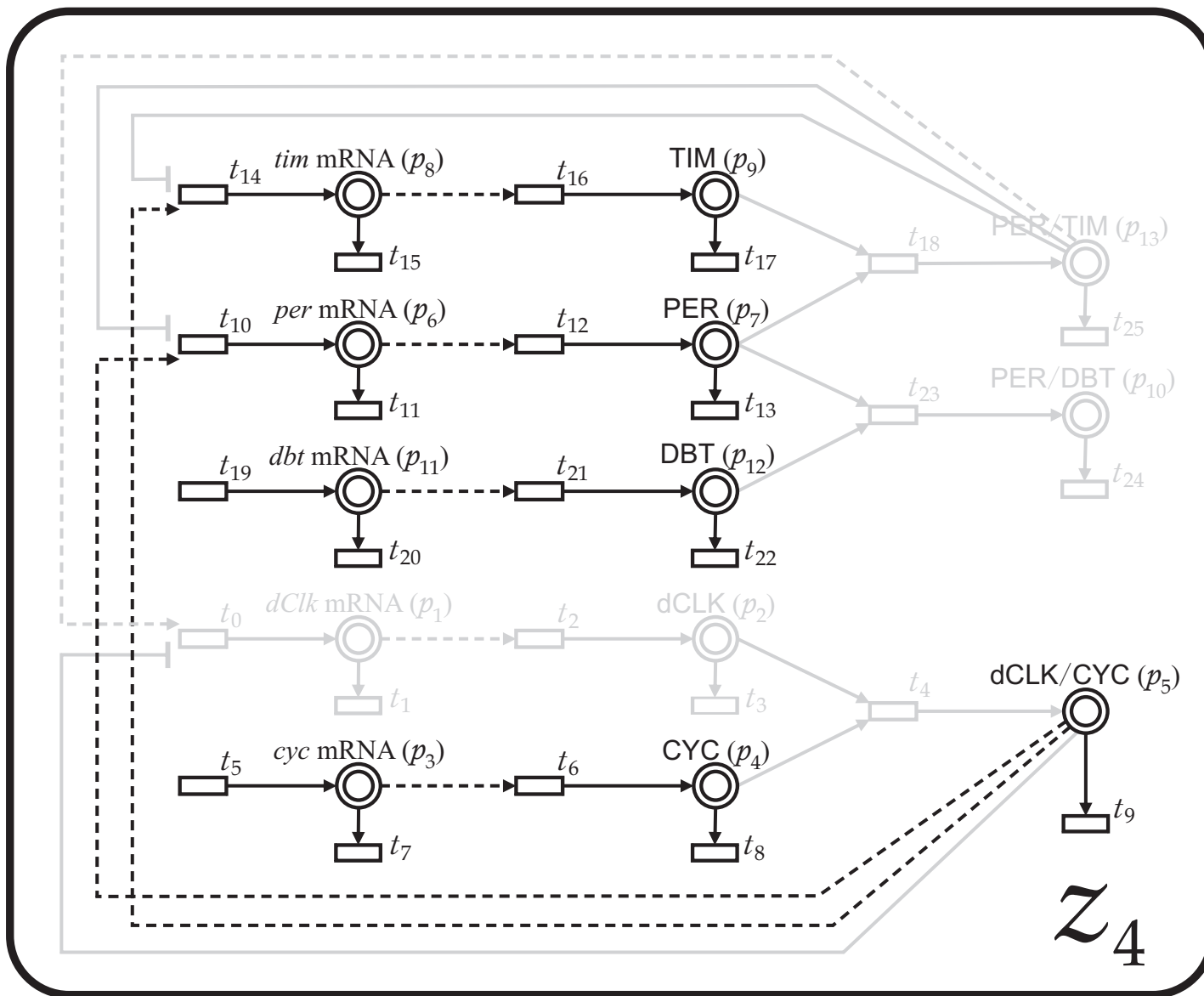

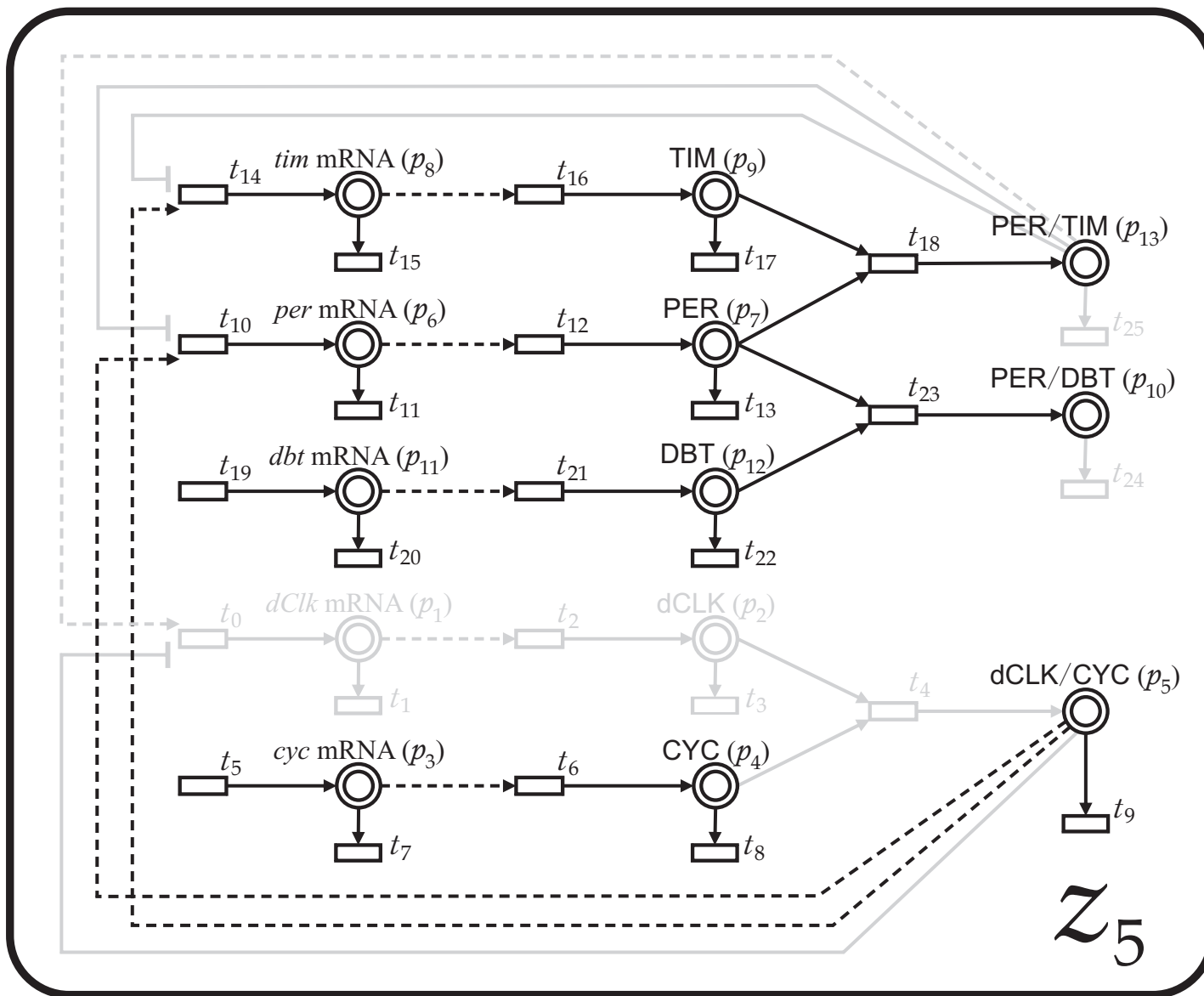

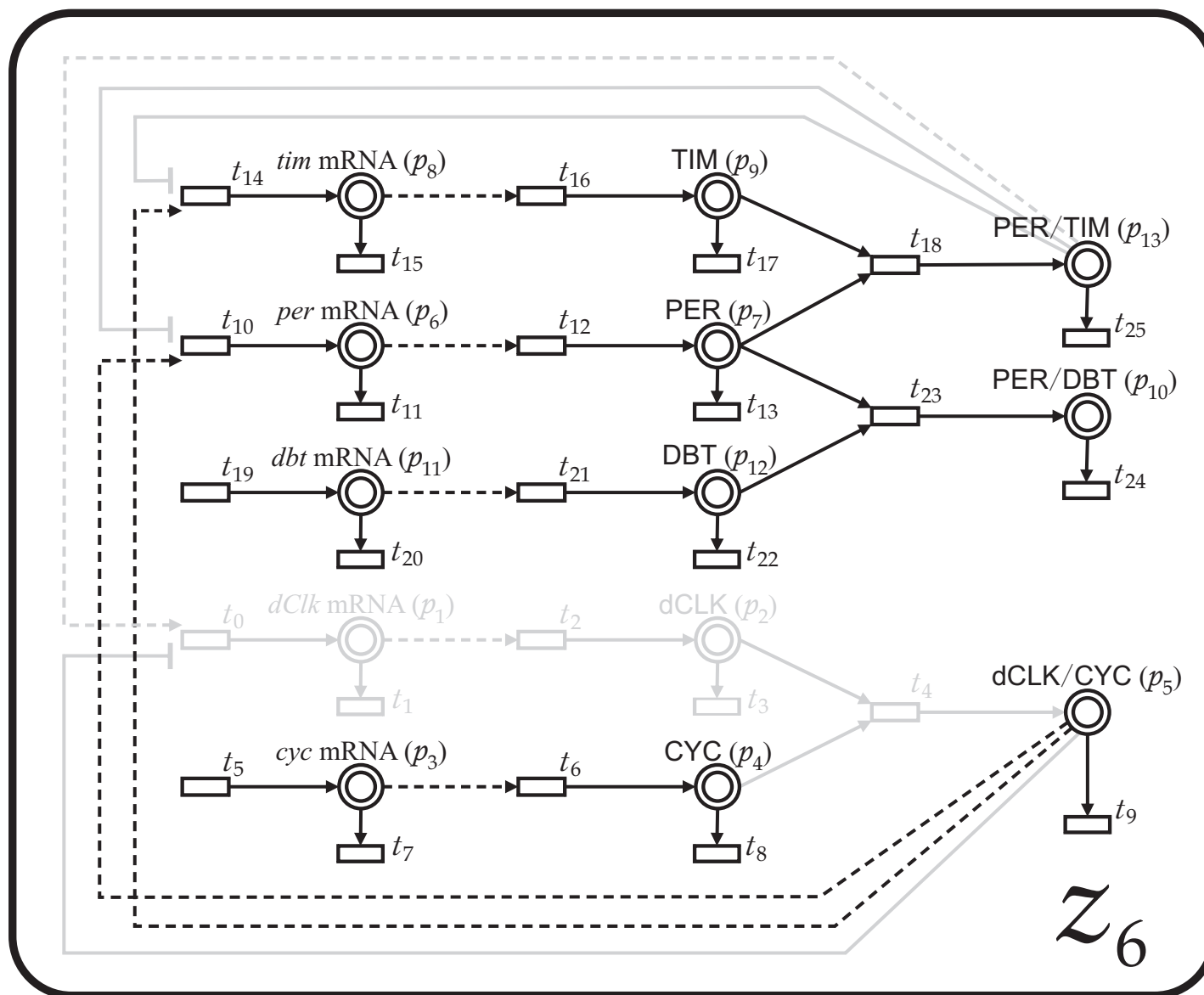

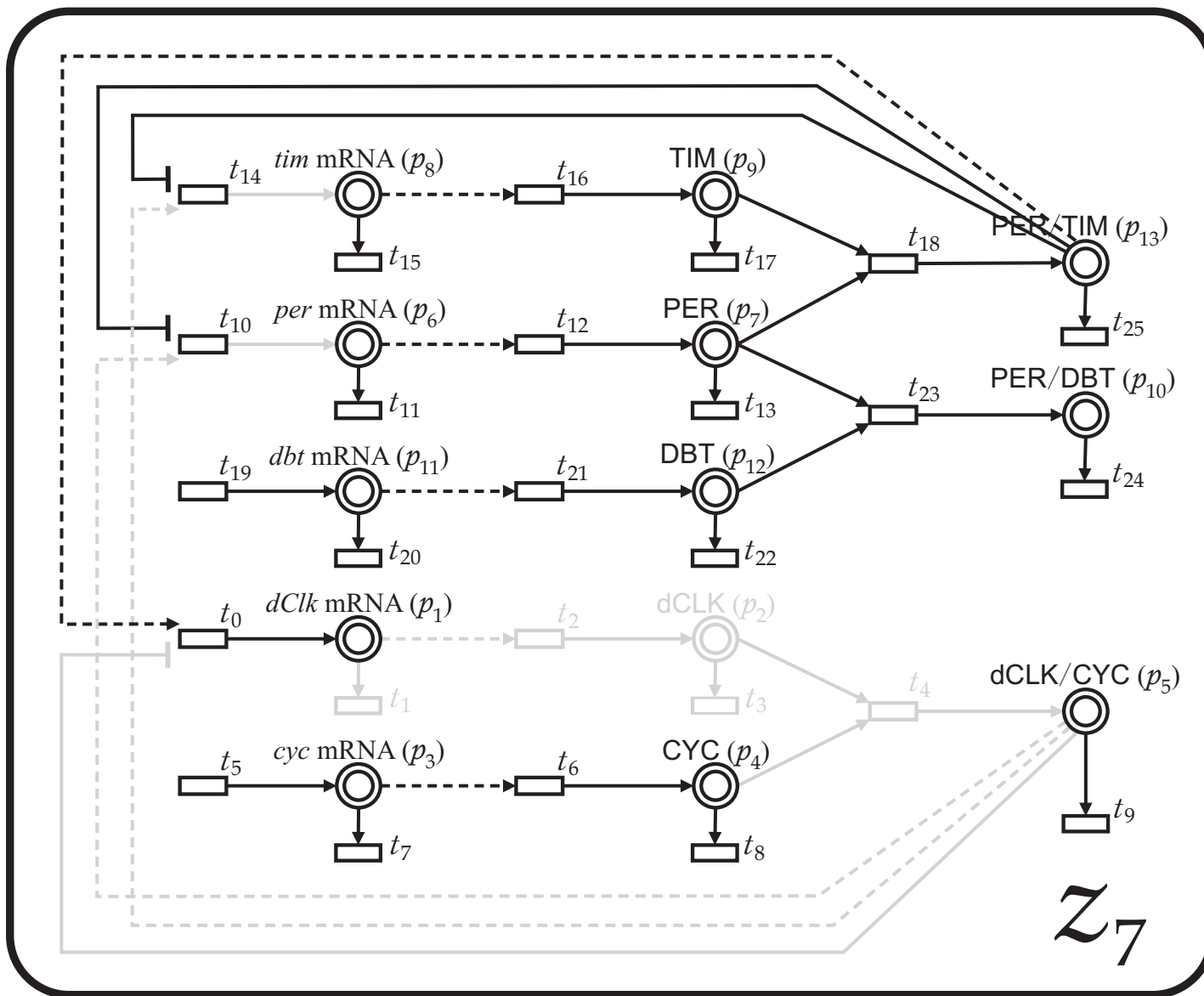

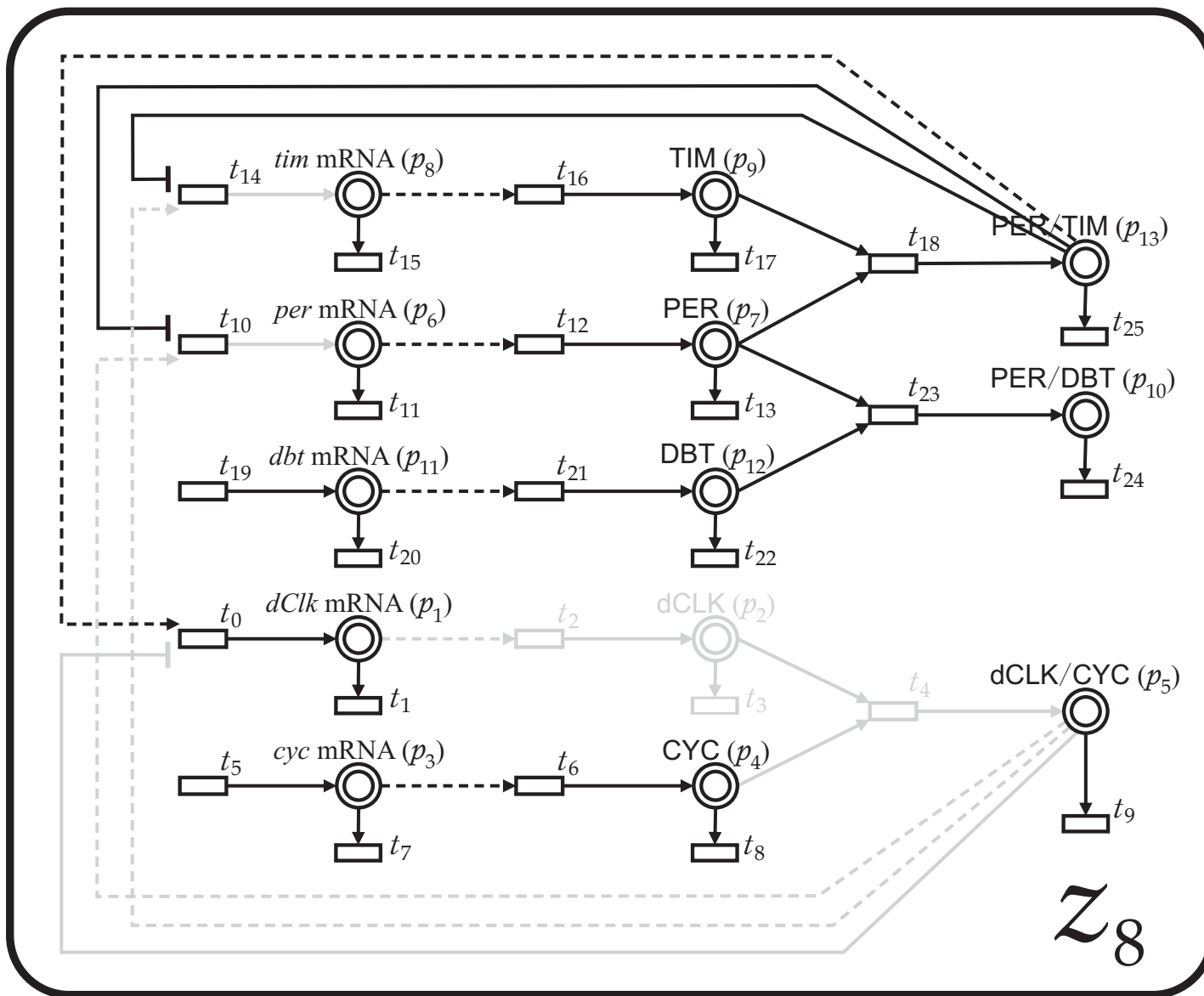

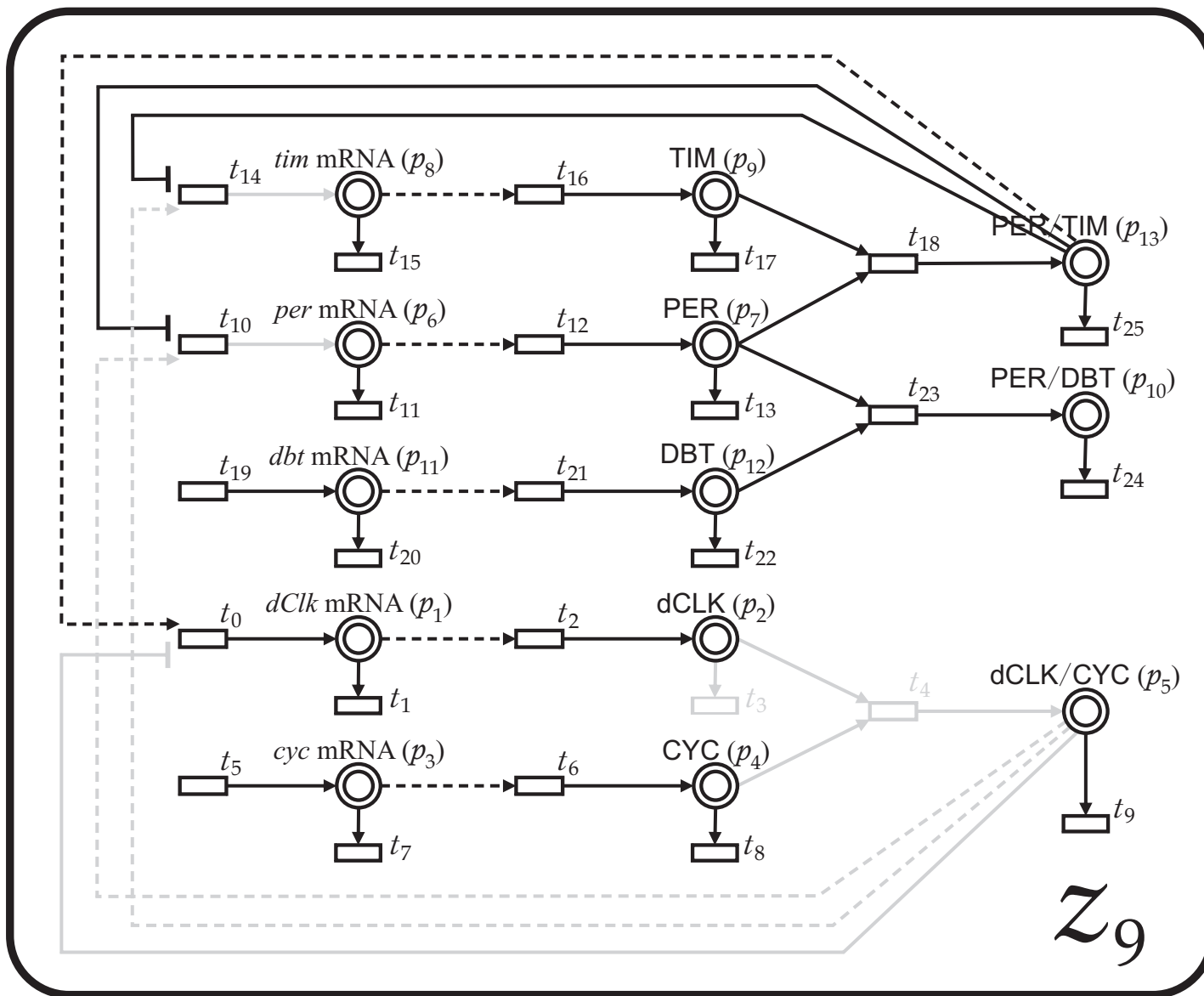

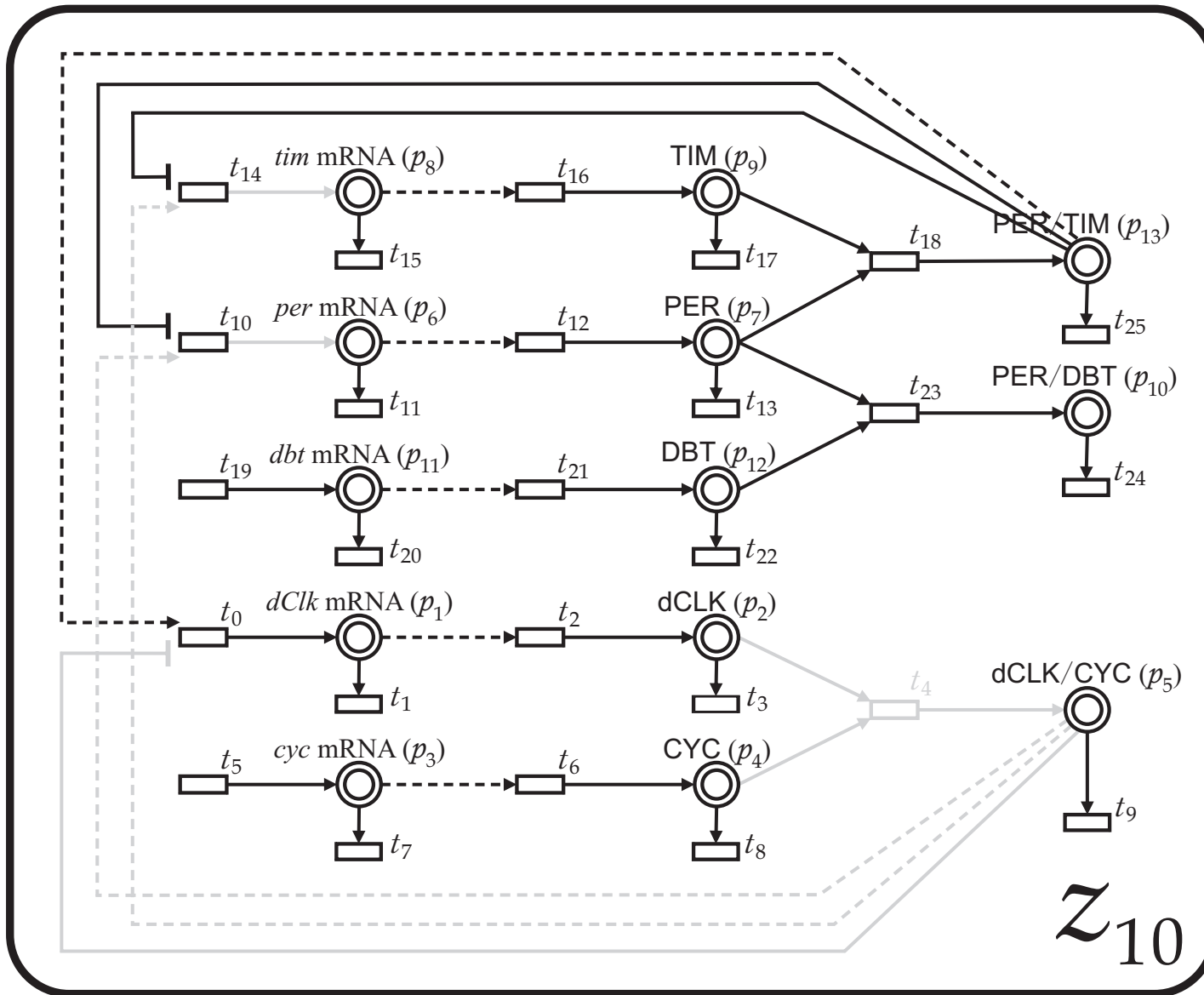

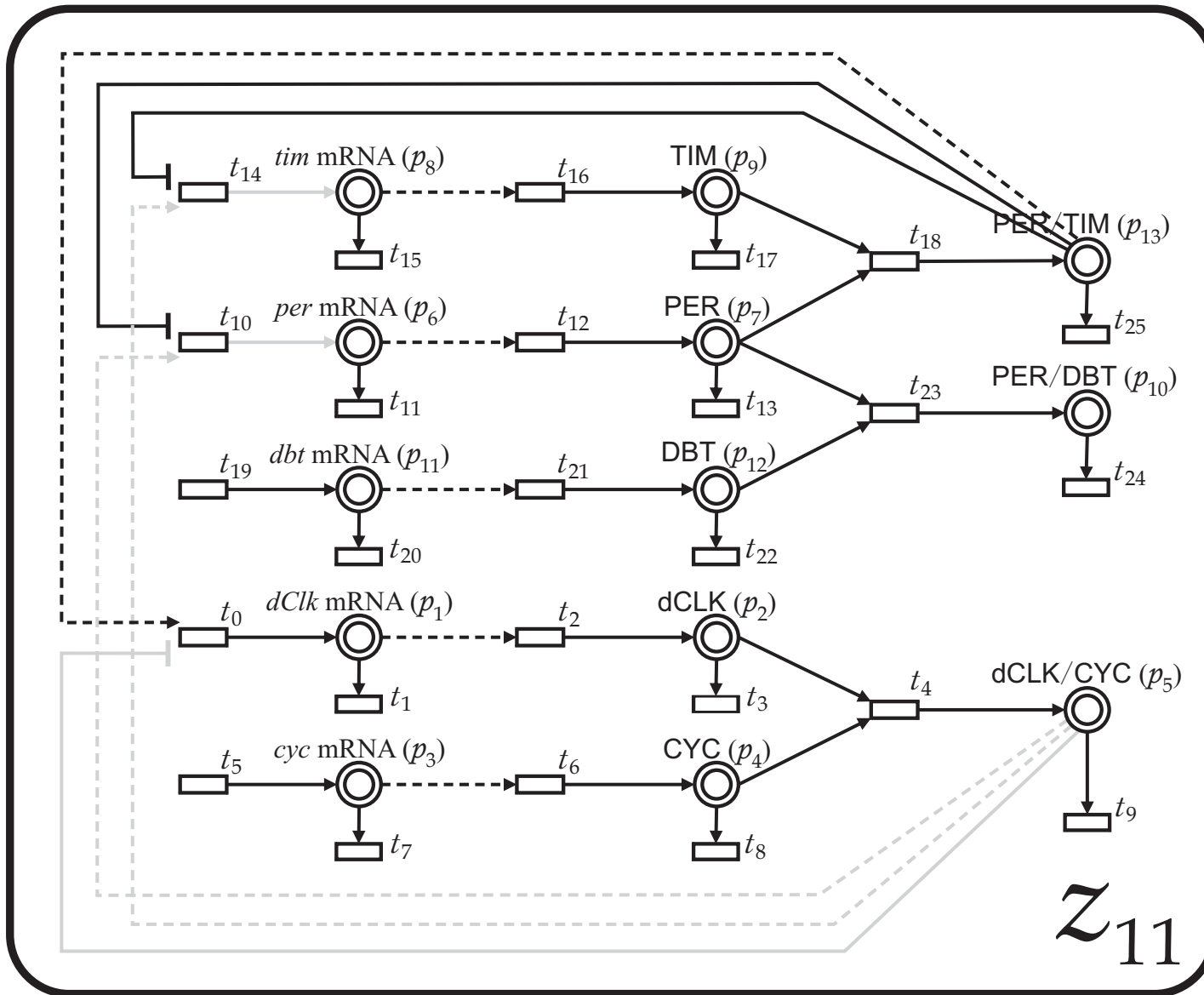

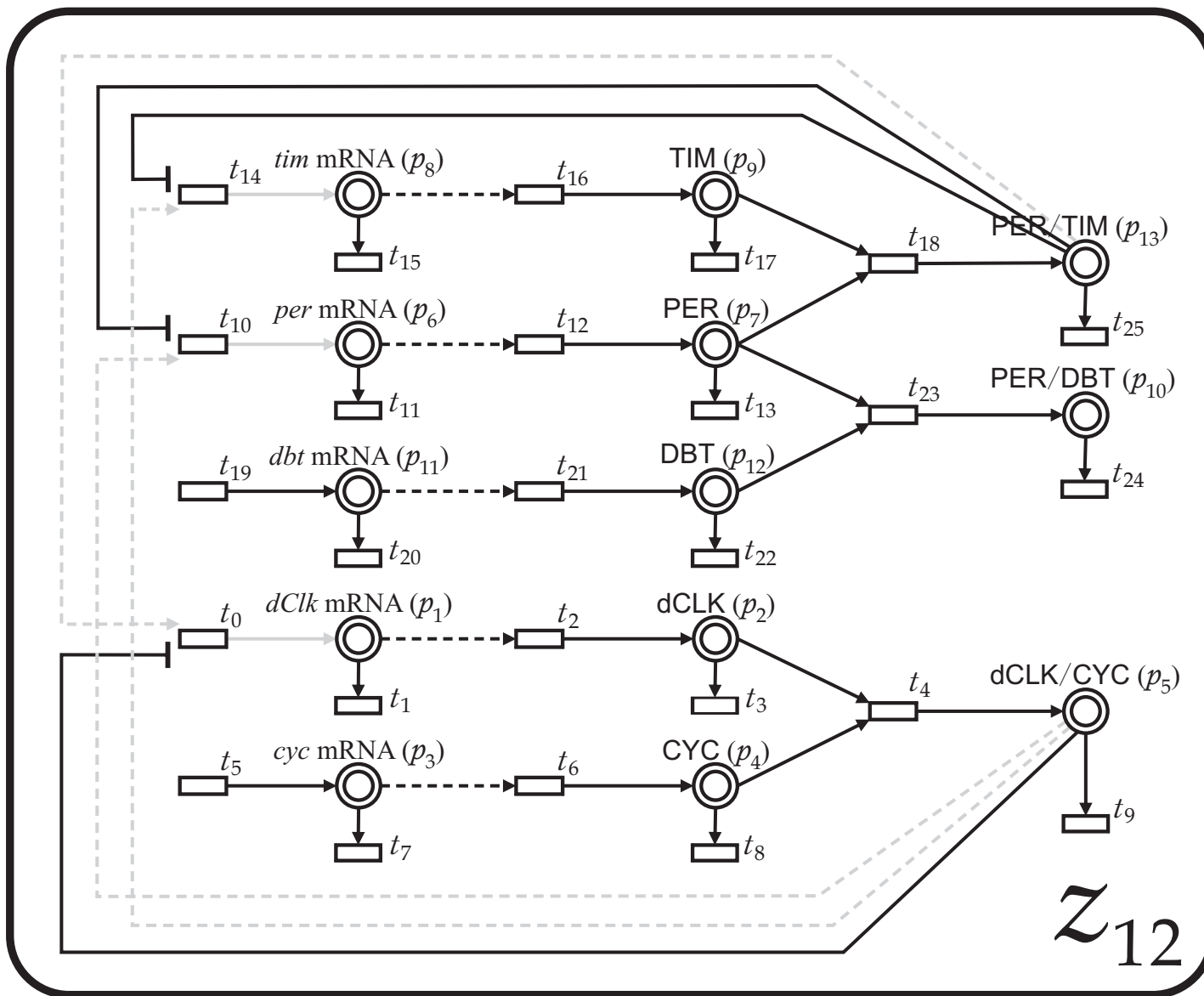

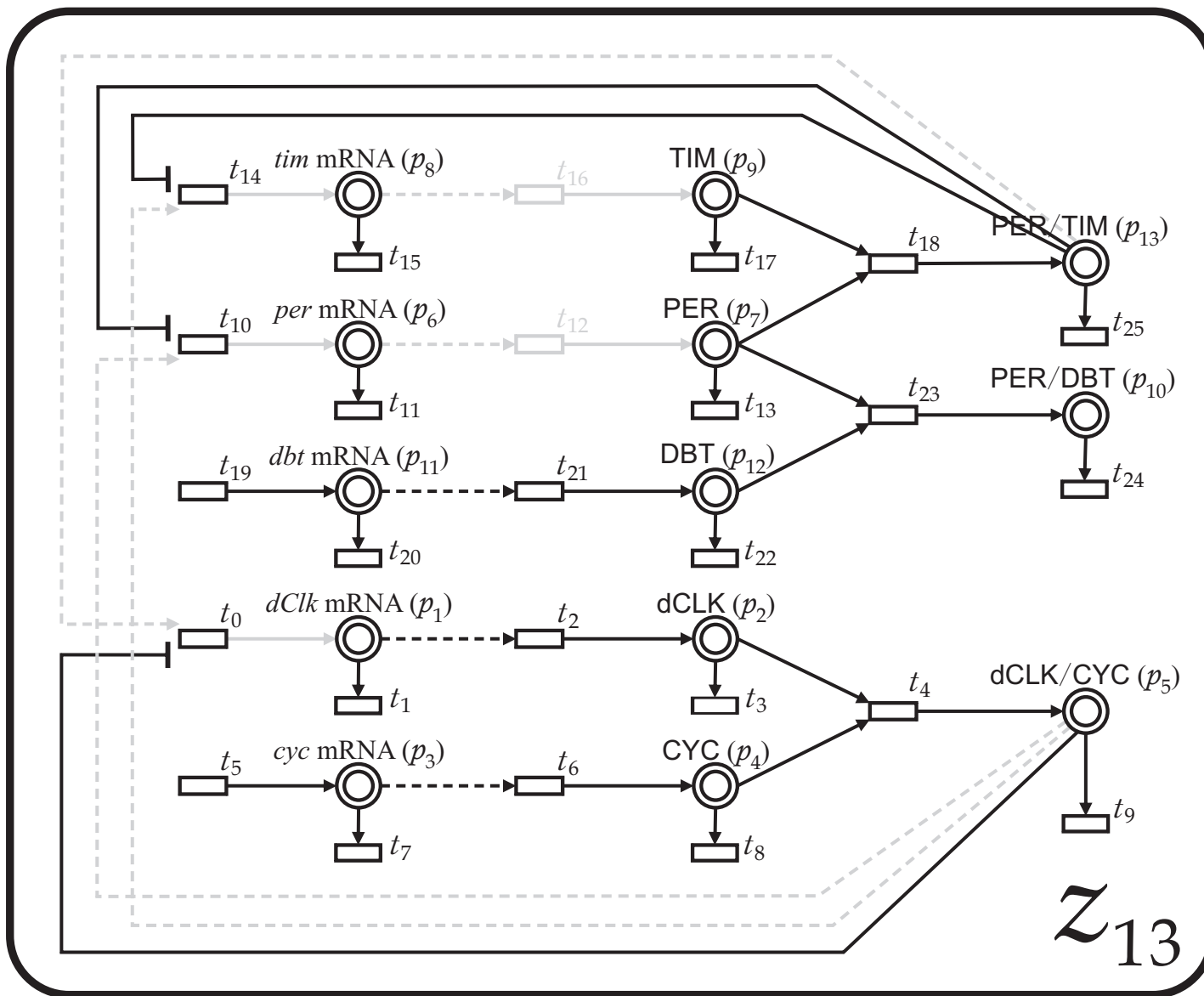

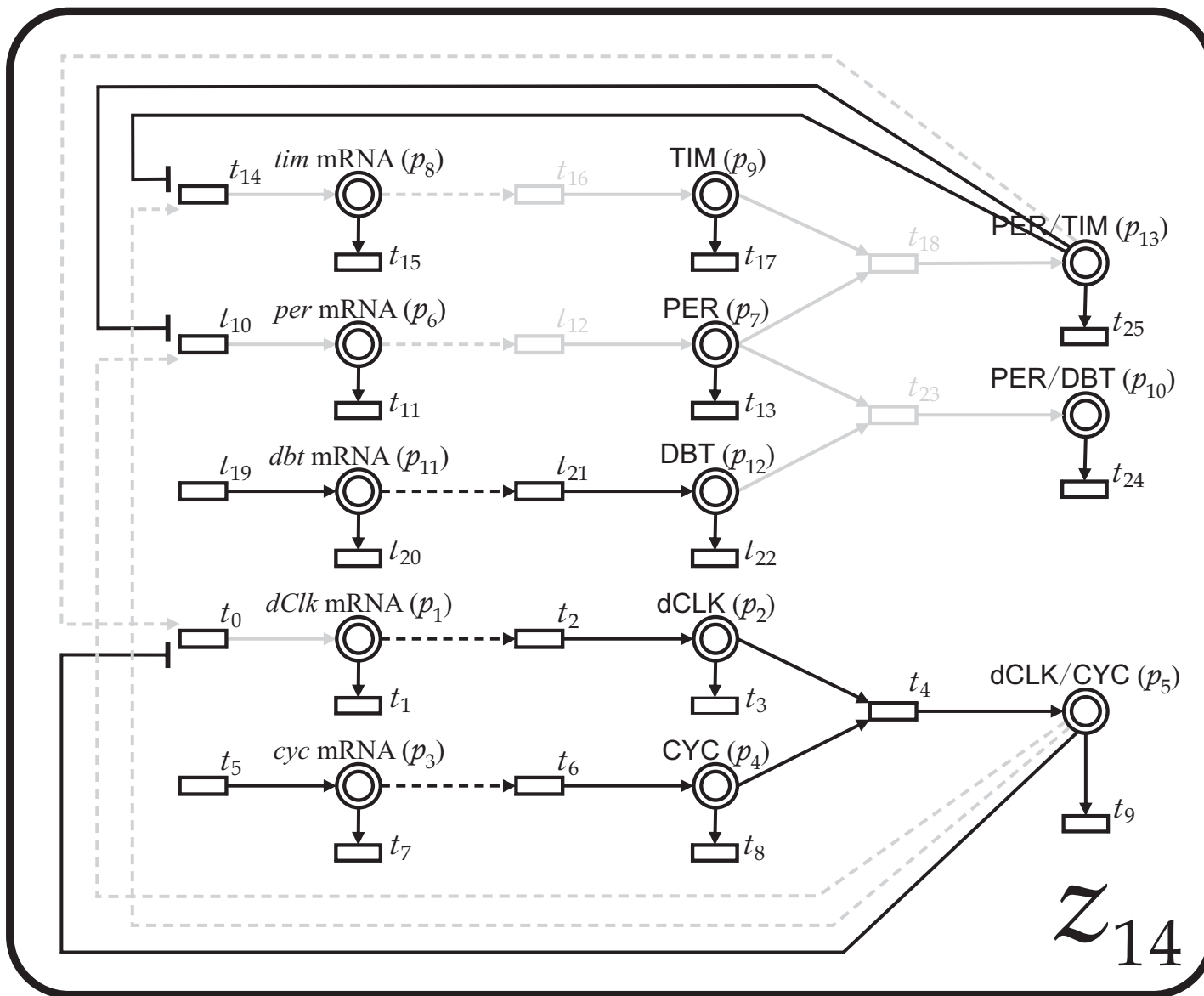

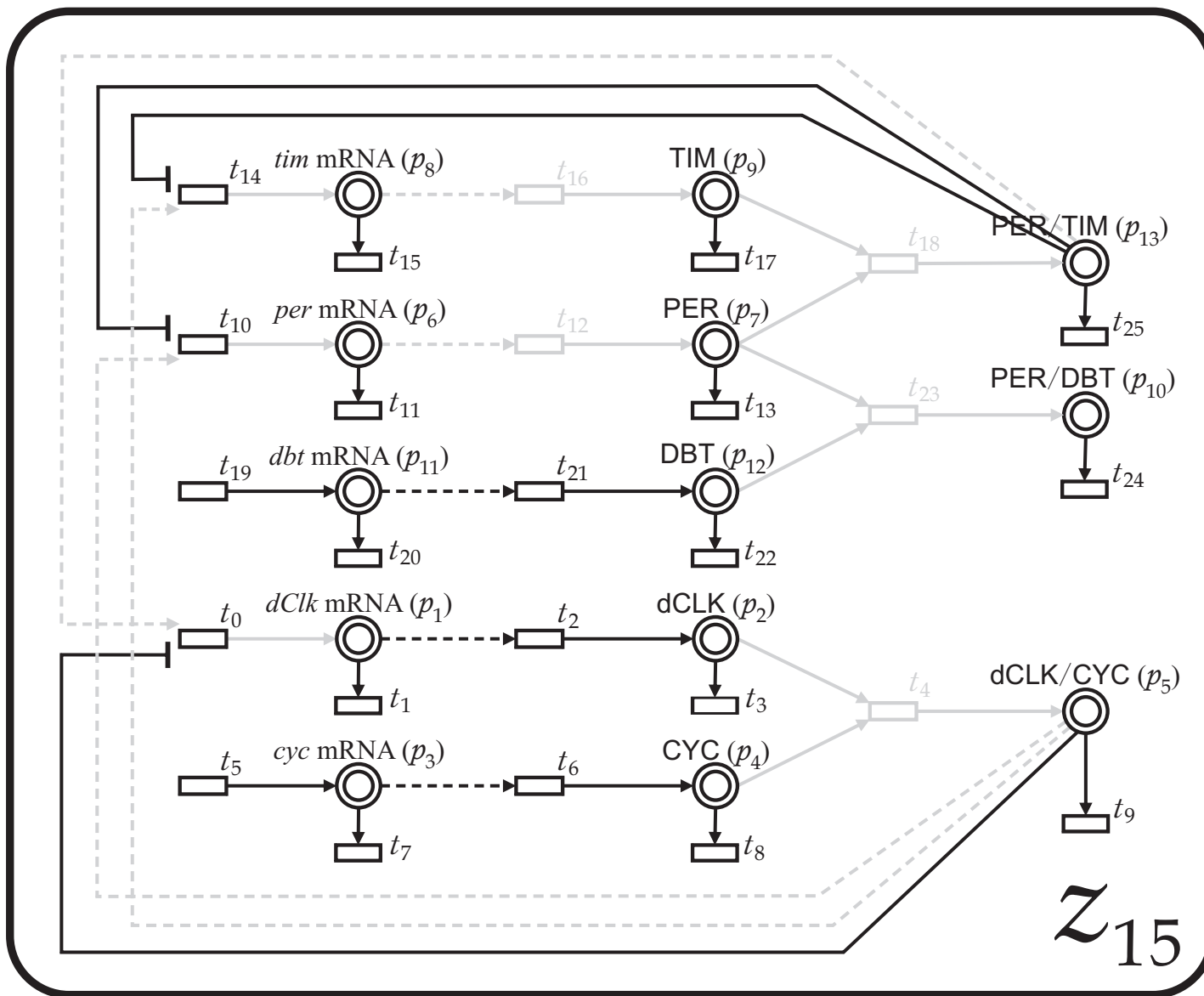

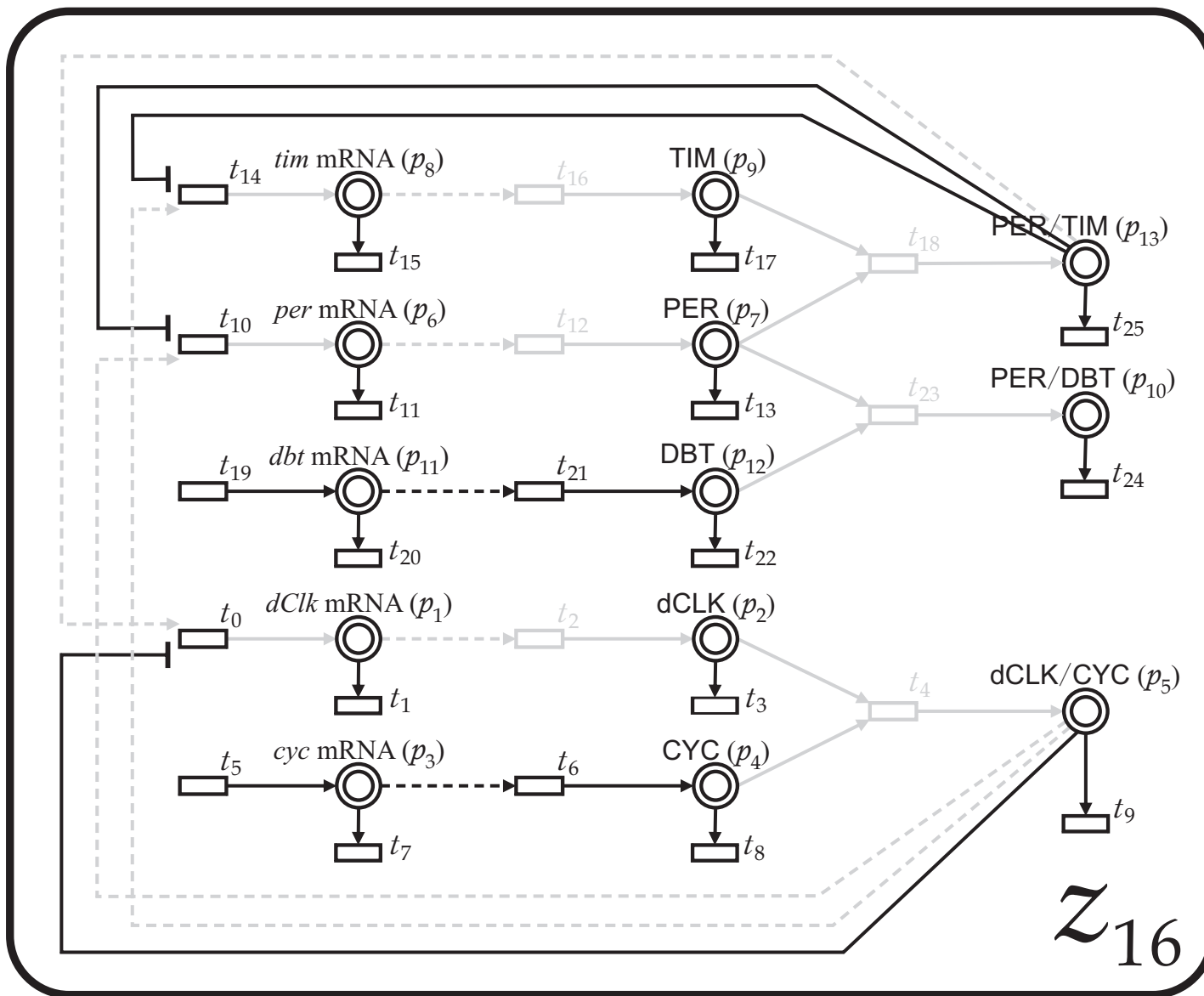

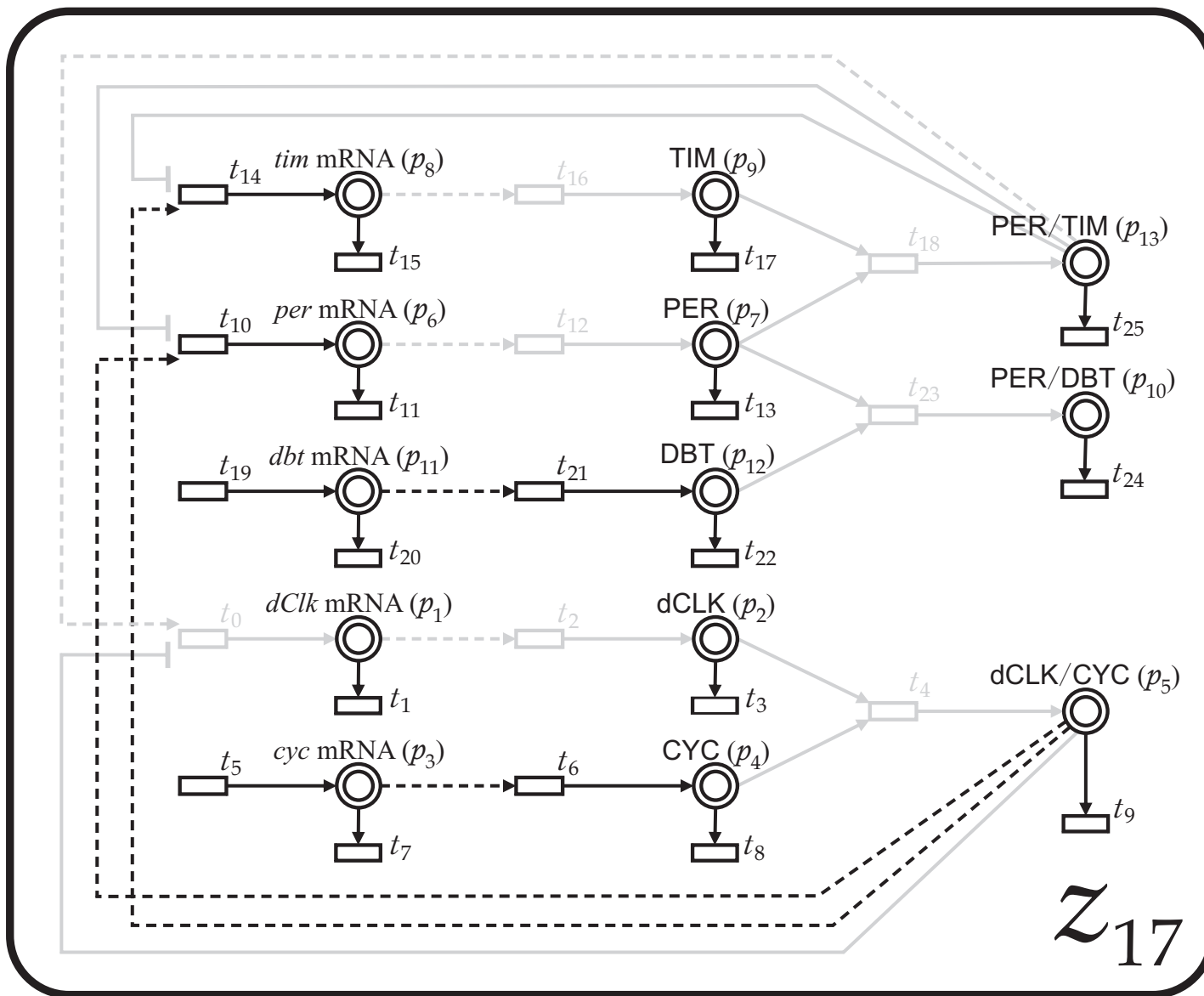

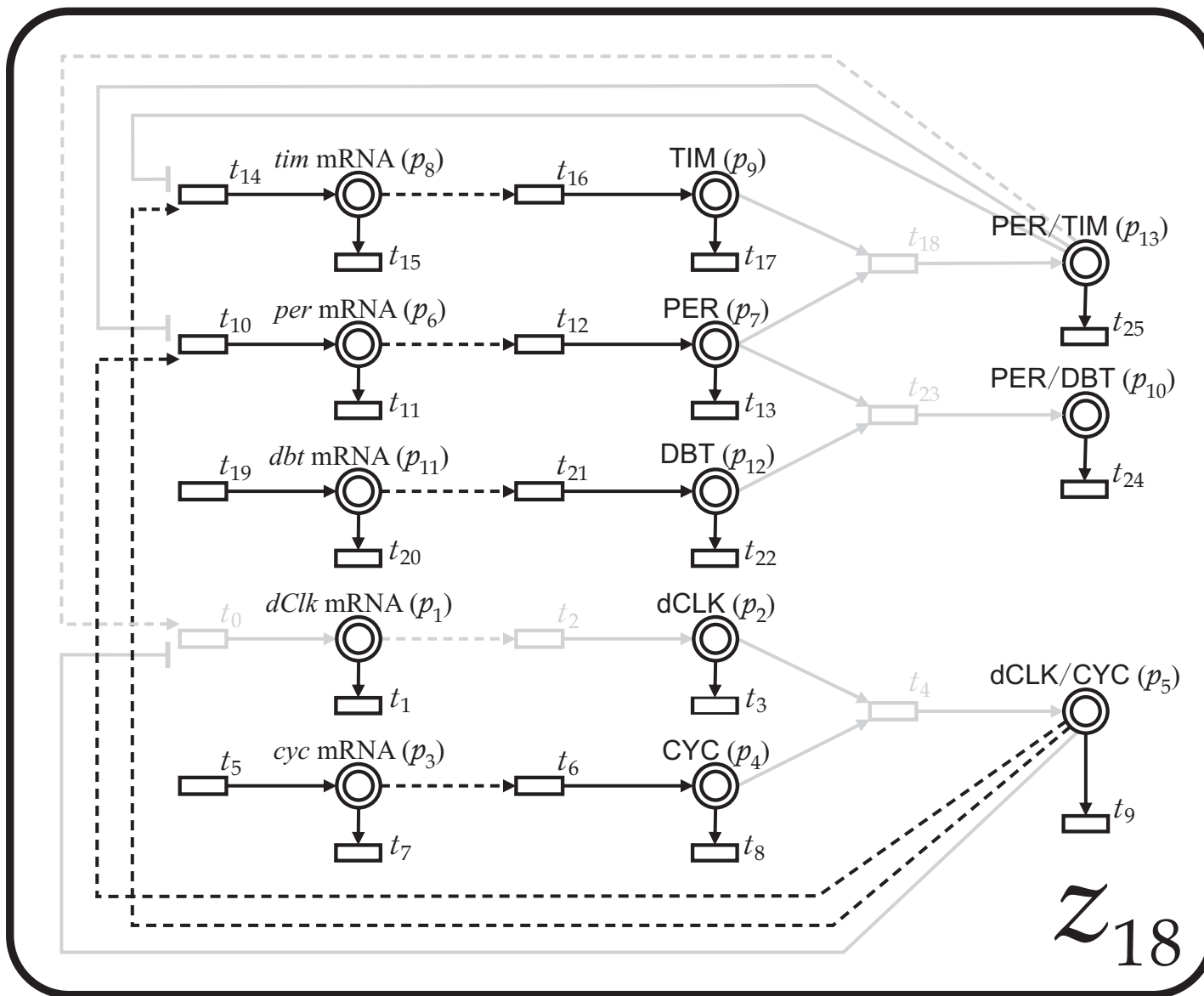

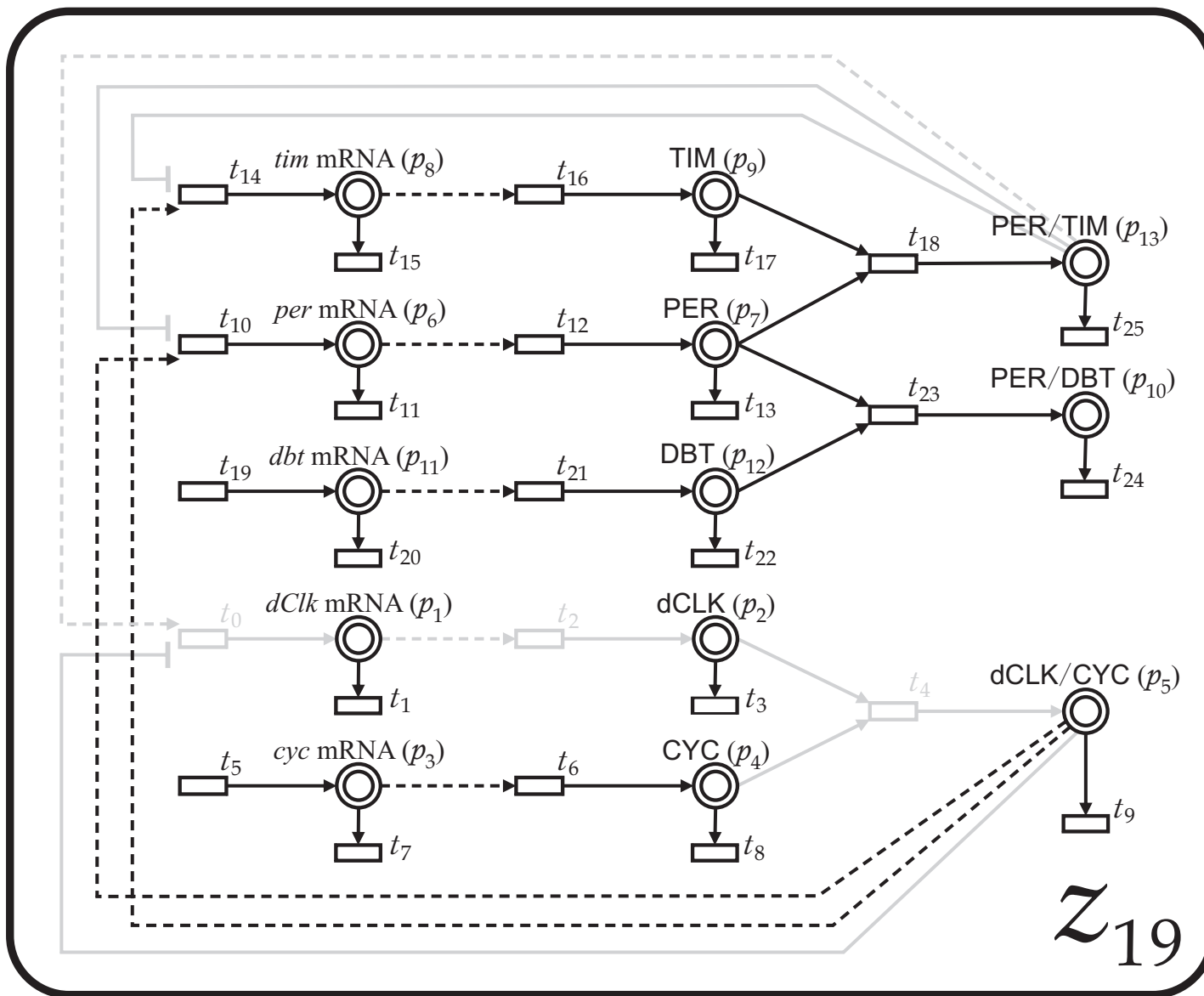

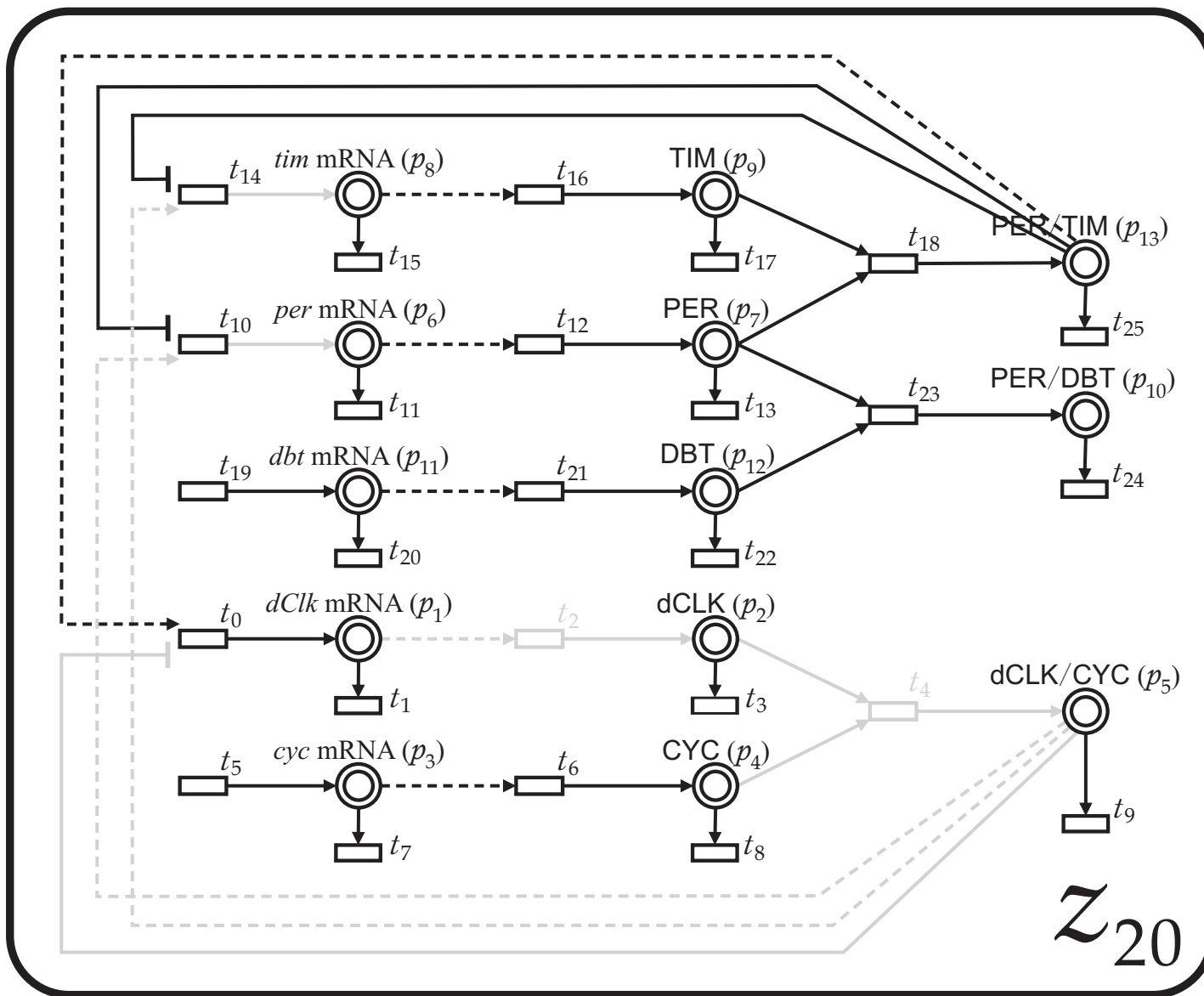

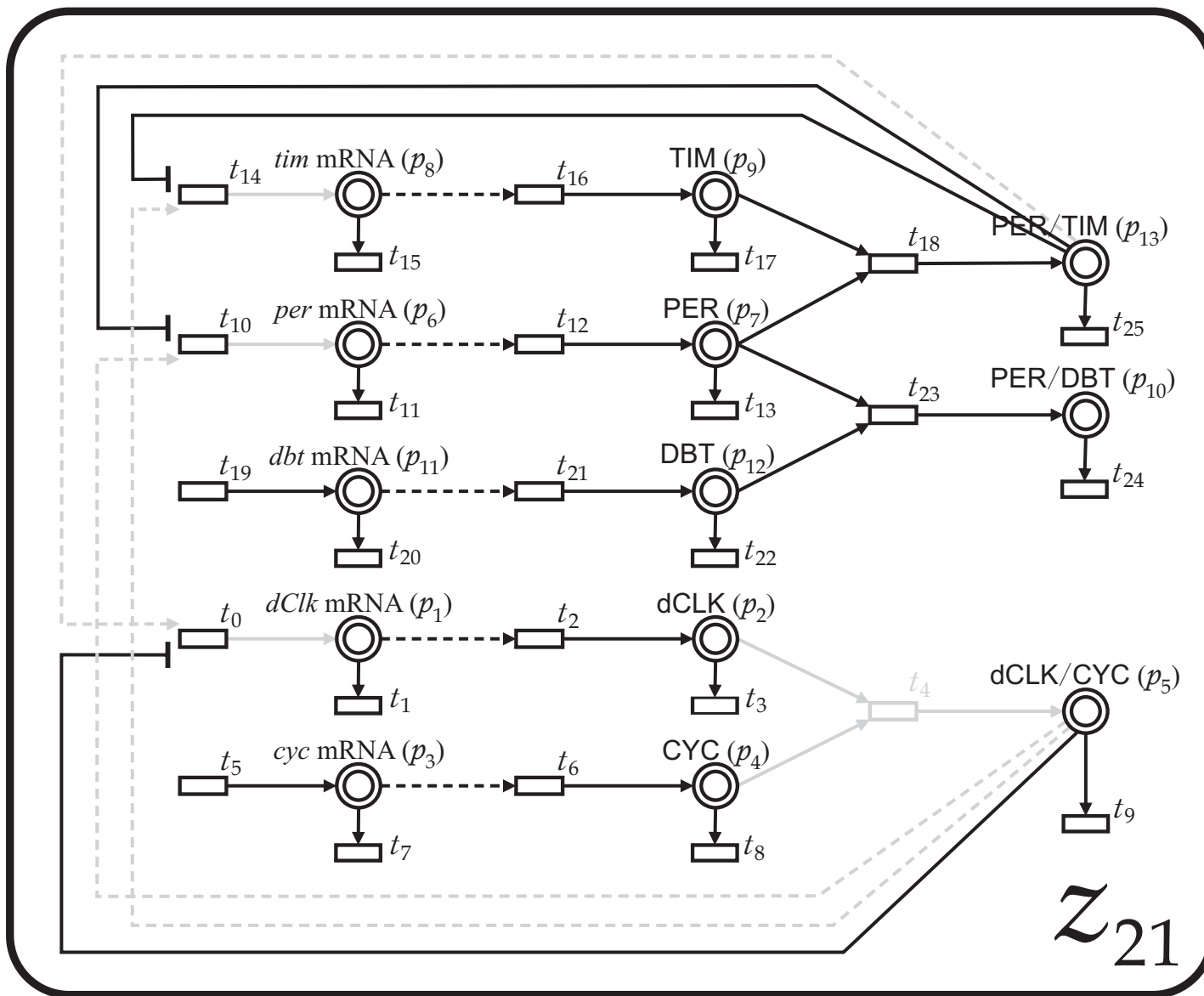

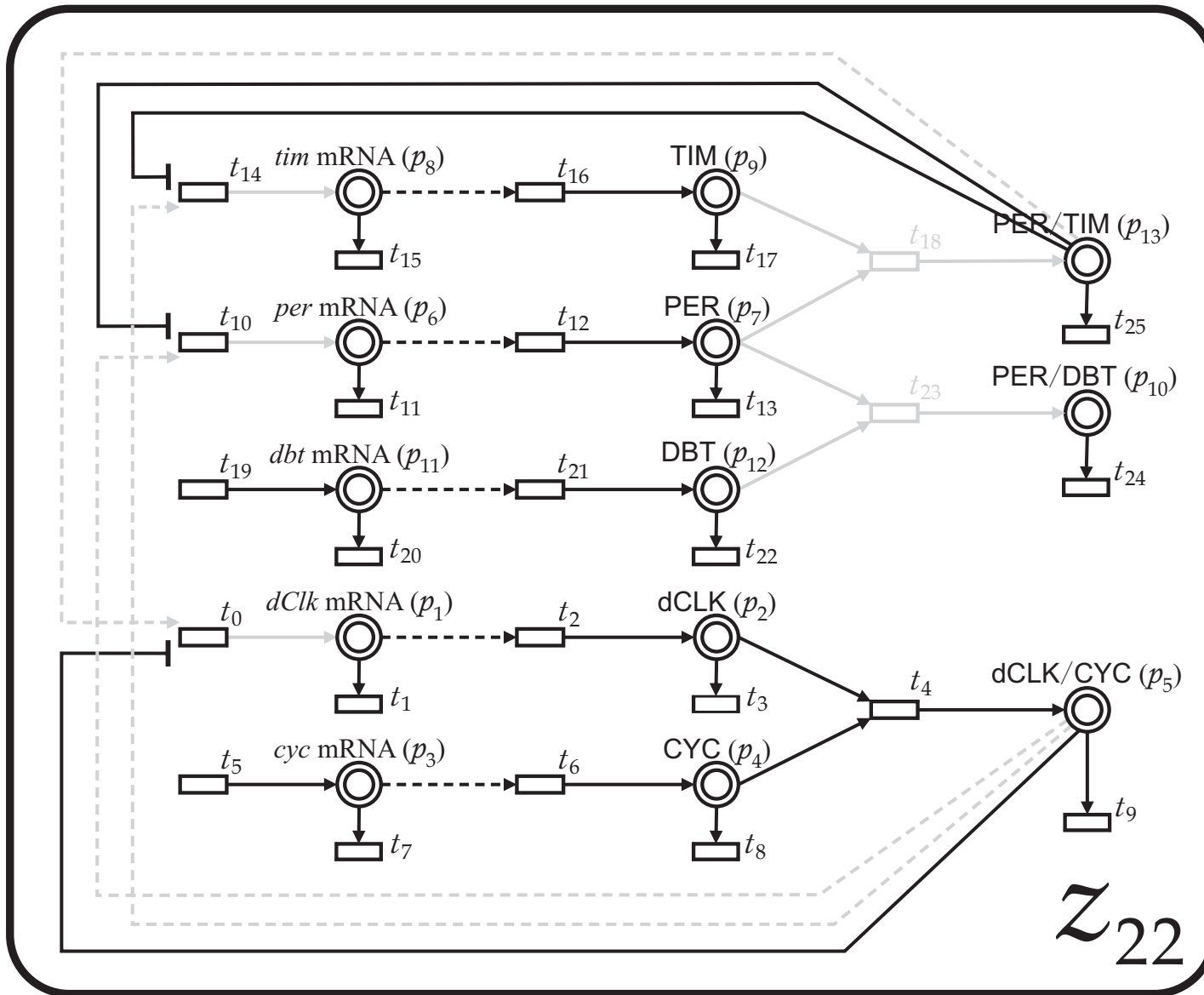

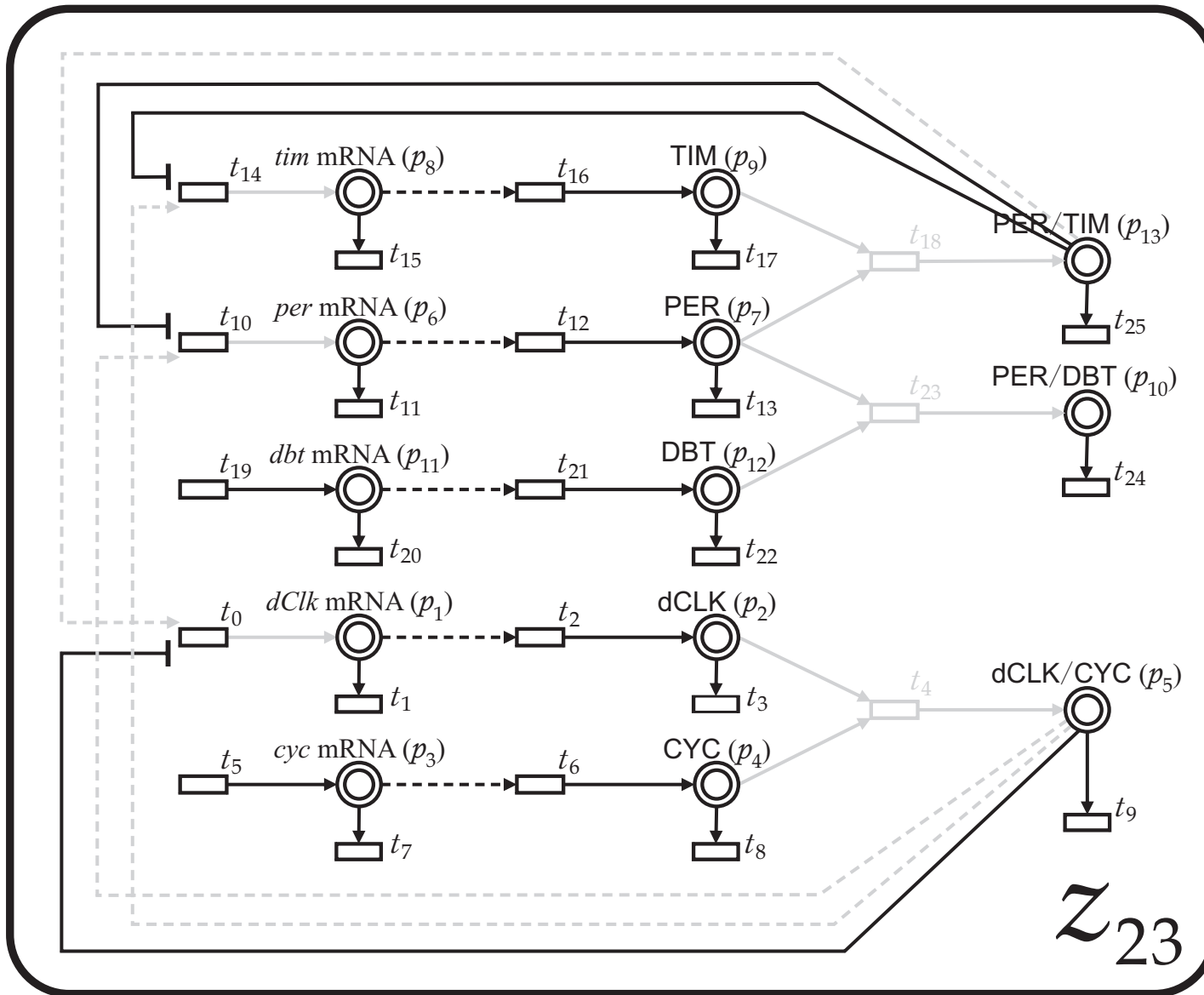

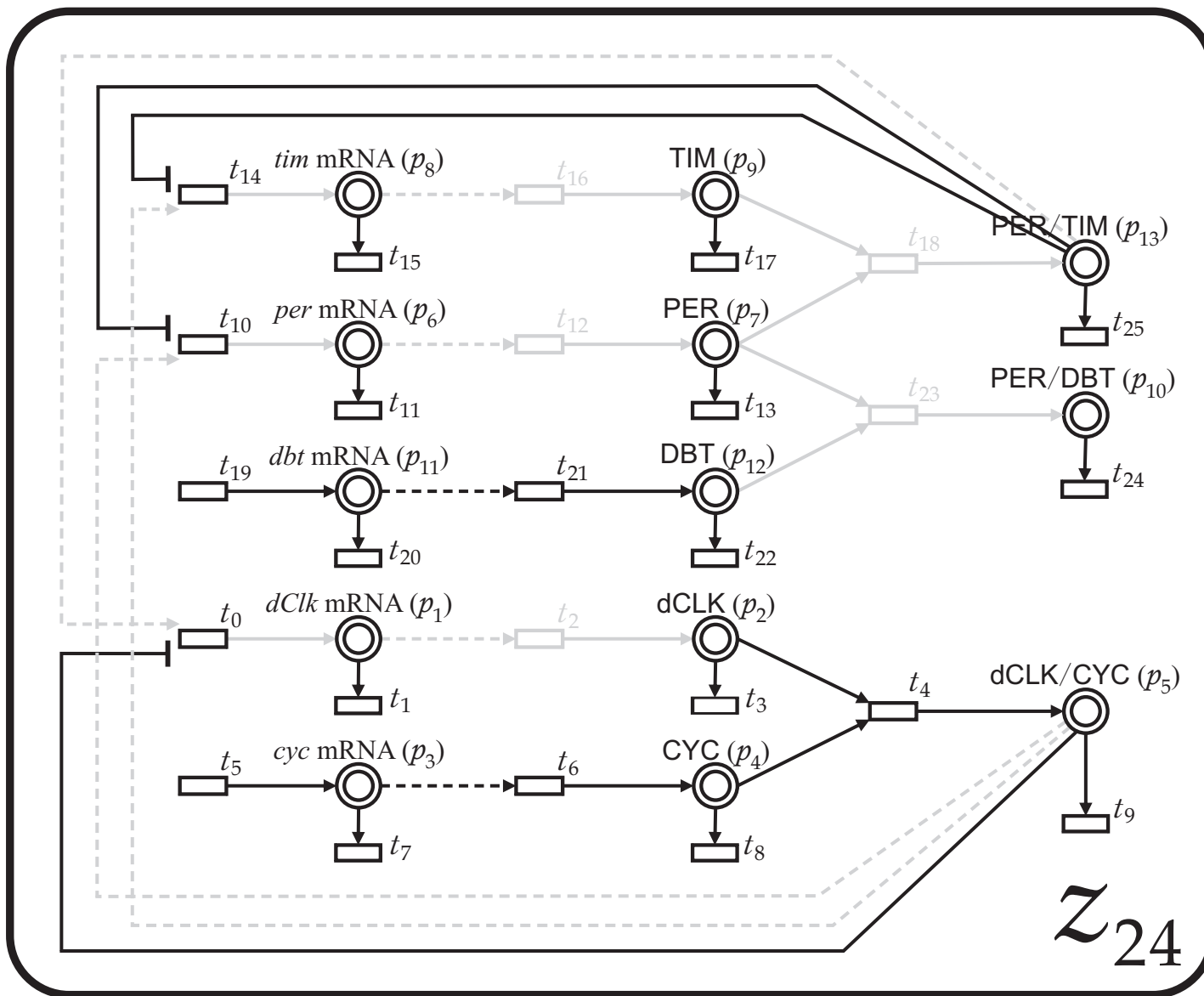

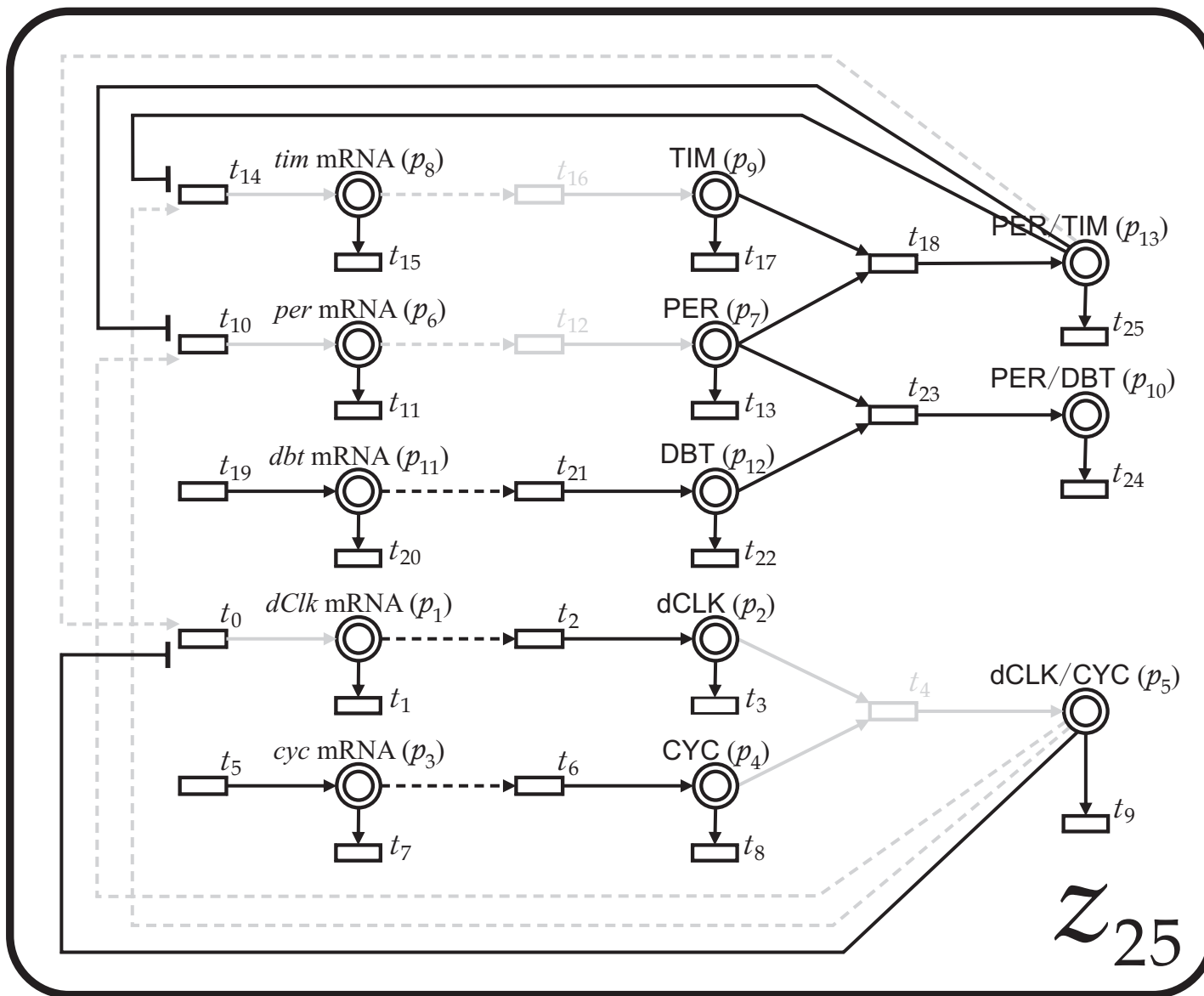

Supplement: Additional file 3 — Detailed net structure of all the nodes in the resulting ASTD. Detailed net structure of all 25 nodes (note that one node is displayed in one page). Readers can turn page forward and back to see the structural difference between two nodes in an easy-to-understand manner. [file 1752-0509-4-39-S3.PDF]

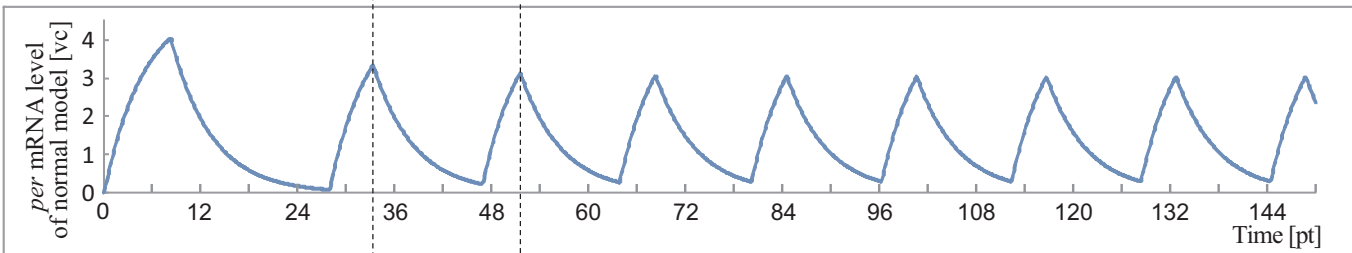

(a)  $m_7 * m_{12} / 85$

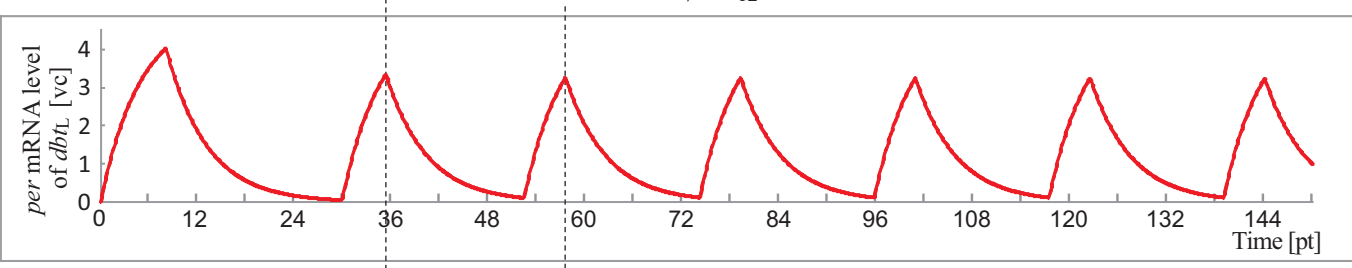

(b)  $m_7 * m_{12} / 1000$

Supplement: Additional file 4 — Concentration behaviors of per mRNA: (a) normal model; and (b) dbtL mutant. Formula such as m7 * m12/1000 for the firing speed of transition t23 is given at charts (a) and (b), which represents complex forming rate of two proteins PER and DBT. The firing speed in dbtL is slower than the one in the normal model. [file 1752-0509-4-39-S4.PDF]
